# Repeated divergence of amphibians and reptiles across an elevational gradient in northern Madagascar

**DOI:** 10.1002/ece3.9914

**Published:** 2023-03-16

**Authors:** Mark D. Scherz, Robin Schmidt, Jason L. Brown, Julian Glos, Ella Z. Lattenkamp, Zafimahery Rakotomalala, Andolalao Rakotoarison, Ricky T. Rakotonindrina, Onja Randriamalala, Achille P. Raselimanana, Safidy M. Rasolonjatovo, Fanomezana M. Ratsoavina, Jary H. Razafindraibe, Frank Glaw, Miguel Vences

**Affiliations:** ^1^ Zoologisches Institut Technische Universität Braunschweig Braunschweig Germany; ^2^ Natural History Museum of Denmark University of Copenhagen Copenhagen Ø Denmark; ^3^ School of Biological Sciences Southern Illinois University Carbondale Illinois USA; ^4^ Institute of Cell and Systems Biology Universität Hamburg Hamburg Germany; ^5^ Neurogenetics of Vocal Communication Group Max Planck Institute for Psycholinguistics Nijmegen The Netherlands; ^6^ Division of Neurobiology, Department of Biology II Ludwig Maximilians University Munich Martinsried Germany; ^7^ Mention Zoologie et Biodiversité Animale Université d'Antananarivo Antananarivo Madagascar; ^8^ School for International Training Antananarivo Madagascar; ^9^ Association Vahatra Antananarivo Madagascar; ^10^ Zoologische Staatssammlung München (ZSM‐SNSB) Munich Germany

**Keywords:** Amphibia, *Brookesia*, *Calumma*, *Mantidactylus*, Montagne d'Ambre National Park, Squamata

## Abstract

How environmental factors shape patterns of biotic diversity in tropical ecosystems is an active field of research, but studies examining the possibility of ecological speciation in terrestrial tropical ecosystems are scarce. We use the isolated rainforest herpetofauna on the Montagne d'Ambre (Amber Mountain) massif in northern Madagascar as a model to explore elevational divergence at the level of populations and communities. Based on intensive sampling and DNA barcoding of amphibians and reptiles along a transect ranging from ca. 470–1470 m above sea level (a.s.l.), we assessed a main peak in species richness at an elevation of ca. 1000 m a.s.l. with 41 species. The proportion of local endemics was highest (about 1/3) at elevations >1100 m a.s.l. Two species of chameleons (*Brookesia tuberculata, Calumma linotum*) and two species of frogs (*Mantidactylus bellyi, M. ambony*) studied in depth by newly developed microsatellite markers showed genetic divergence up the slope of the mountain, some quite strong, others very weak, but in each case with genetic breaks between 1100 and 1270 m a.s.l. Genetic clusters were found in transect sections significantly differing in bioclimate and herpetological community composition. A decrease in body size was detected in several species with increasing elevation. The studied rainforest amphibians and reptiles show concordant population genetic differentiation across elevation along with morphological and niche differentiation. Whether this parapatric or microallopatric differentiation will suffice for the completion of speciation is, however, unclear, and available phylogeographic evidence rather suggests that a complex interplay between ecological and allopatric divergence processes is involved in generating the extraordinary species diversity of Madagascar's biota. Our study reveals concordant patterns of diversification among main elevational bands, but suggests that these adaptational processes are only part of the complex of processes leading to species formation, among which geographical isolation is probably also important.

## Graphical abstract

We use the isolated rainforest herpetofauna on the volcanic Montagne d'Ambre (Amber Mountain) massif in northern Madagascar as a model to explore elevational divergence at the level of populations and communities. Based on intensive sampling and DNA barcoding of amphibians and reptiles along an elevational transect, we assessed a main peak in species richness at an elevation of ca. 1000 m a.s.l. with 41 species, and genetic divergences in four target species with turnover occurring between 1100 and 1270 m above sea level. Our data illustrate that species of rainforest amphibians and reptiles in Madagascar show concordant population genetic differentiation across elevation, along with morphological and elevational differentiation, and in partial agreement with patterns of community turnover.

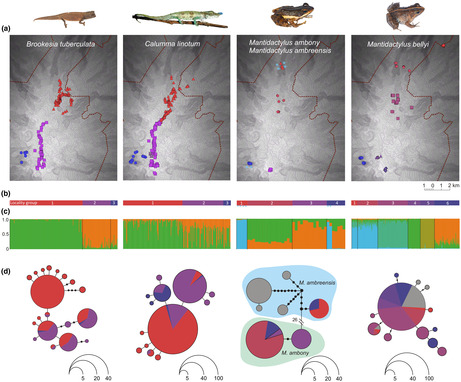

## 1 INTRODUCTION

The processes by which new species arise have long been an active field of debate. Mayr (1942, 1947), and others made a case for prevalence of allopatric (vicariant) speciation, which relies on the assumption that intrinsic barriers to gene flow were unlikely to evolve in the absence of extrinsic barriers such as geographic obstacles (Fitzpatrick et al., 2009). This claim, however, has been challenged by numerous intriguing examples of sympatric diversification in a large variety of organisms, often triggered by ecological adaptation, sometimes leading to complete speciation (e.g. Arnegard et al., 2014; Bush, 1969; Hernández‐Hernández et al., 2021; Kautt et al., 2020; Schliewen et al., 1994; Steinfartz et al., 2007). In fact, the focus of speciation research has for some time now shifted to focus less on the spatial dimension and more on adaptive vs. non‐adaptive mechanistic drivers (Butlin et al., 2008; Via, 2002), and the underlying genomics (e.g. Feder et al., 2013; Kautt et al., 2020; Seehausen et al., 2014).

Where species formation proceeds without geographic barriers to gene flow, sexual or ecological selective mechanisms are typically invoked to trigger and maintain divergence. Ecological speciation occurs in any geographical context, if natural selection strongly favors different ecotypes, and reproductive isolation evolves as a consequence of adaptive divergence due to differing ecology (Dieckmann et al., 2004; Nosil, 2012; Schluter, 2001). Striking examples of this mechanism come from lake or island systems, often with fascinating repeated origins of similar adaptations (e.g. de León et al., 2010; Kautt et al., 2020; Losos, 2009; McKinnon et al., 2004). A recent review found that ecological speciation is prevalent across the tree of life, often associated with abiotic habitat factors such as temperature (Hernández‐Hernández et al., 2021).

Mountain massifs have long been considered as cradles for diversification of organisms (Stebbins, 1974). For instance, mountains provide the geographical substrate for vicariant divergence on certain elevational bands, creating ecological gradients that become horizontally separated, across mountains, through climatic or topographic change, followed by allopatric divergence in these refugia – as an example, in the coastal montane complex of South America, only few montane amphibian and reptile taxa are shared among massifs while a higher proportion of shared biodiversity was observed for taxa occurring at lower elevations (Rivas et al., 2021). In addition, mountains also contain steep elevational gradients and ecotones allowing for adaptive divergence in parapatric settings (Hall, 2005). That ecotones can trigger adaptive divergence has been known since the pioneering study of Schneider et al. (1999), and some authors hypothesized that parapatric divergence across elevational gradients can lead to completed species formation (e.g. Hall, 2005; Linck et al., 2020). However, other case studies found partial but not full reduction of gene flow across elevation (e.g. Bachmann et al., 2020; Funk et al., 2016), suggesting that this mechanism may often be insufficient to yield fully separated species.

Although studies on fish radiations have probably produced the most convincing examples of ecological speciation, numerous important studies have also used amphibians and reptiles as models for processes of ecological divergence (Wollenberg Valero et al., 2019). Because the majority of amphibian and reptile species are terrestrial, relatively small‐sized, and have limited dispersal ability, they are known to diverge adaptively across ecotones (Rosenblum & Harmon, 2011; Schneider et al., 1999), elevational gradients (Bachmann et al., 2020; Funk et al., 2016), and breeding habitat types (Steinfartz et al., 2007). Completion of species formation by such adaptive mechanisms has so far not been demonstrated in amphibian or reptile systems, and hybrid zone analyses suggest a role for gradual, likely non‐adaptive accumulation of barrier loci in frogs (Dufresnes et al., 2021). Yet, the existence of repeated ecomorph evolution in anoles (Losos, 2009) and existence of young, sympatric sister species pairs in frogs (Vences et al., 2012) suggest a need for further exploration of ecological speciation in these organisms.

Among regions of high organismal diversity and regional endemism in the tropics, Madagascar, the fourth largest island of the world, has often been cited as the “hottest hotspot” globally (Ganzhorn et al., 2001). The uniqueness and diversity of Madagascar's biota, with high proportions of microendemic species (Wilmé et al., 2006), has been explained by various diversification mechanisms (reviewed in Vences et al., 2009), a combination of which likely produced the current species patterns on the island (Brown et al., 2014). Especially in northern Madagascar, the high elevational heterogeneity has likely been causal for high rates of speciation (Brown et al., 2016), which Raxworthy and Nussbaum (1995) suggested were caused by vicariance in montane refugia. Within this part of Madagascar, one volcanic Massif, the Amber Mountain or Montagne d'Ambre, stands out due to its isolation from the main eastern rainforest band. Surrounded by drier biomes, and characterized by local endemism as well as steep elevational gradients, Montagne d'Ambre is an excellent geographic region to test hypotheses of ecological speciation in Madagascar's herpetofauna.

Here, we undertake a multi‐taxon approach to better understand the role of elevational gradients in diversification and species formation at Montagne d'Ambre. From a dense transect sampling, we combine (i) population genetic data from four species (complexes) of frogs and chameleons, (ii) high‐resolution modeling of bioclimatic niches of genetic clusters identified in these lineages, and (iii) comparisons of species richness and turnover across the transect, based on an extensive set of DNA barcoded samples.

With these data, we set out to answer four core questions: (1) Is there a continuous transition in community composition up the slope of Montagne d'Ambre, or are there any marked breaks in reptile and amphibian species assemblages? Major elevational turnover in community assemblage would provide strong evidence that the ecotone is ecologically significant. (2) Do individual species (complexes) found across the elevational gradient show evidence of continuous gene‐flow, or is there genetic substructure that coincides with the ecotone? And if genetic substructure exists, does it (3) correlate with morphological or ecological niche differences, and/or (4) coincide among different taxa and with overall community turnover? Aligned, discontinuous genetic variation across an ecologically significant ecotone would provide evidence for a significant role of this ecotone in generating and/or maintaining diversity.

## 2 MATERIALS AND METHODS

### 2.1 Sampling locality and field methods

This study focuses on four main target species (complexes) that occur over a wide elevational range at Montagne d'Ambre: two chameleons (*Calumma linotum*, *Brookesia tuberculata*) and two mantellid frogs (*Mantidactylus bellyi*, and the complex of *M. ambreensis* and *M. ambony*, which we taxonomically resolved only after the fieldwork was conducted and partly based on it; Scherz et al., 2020). All other amphibians and reptiles encountered during our survey were recorded and genetically identified for general analysis of community structure. A full list of all species and georeferenced records is included in the Figshare repository (DOI 10.6084/m9.figshare.14618175).

The main fieldwork involved in this work was conducted between November 2017 and January 2018 (from the start to the peak of the rainy season) on Montagne d'Ambre, a volcanic massif in northernmost Madagascar (located between 12.34–12.75° S and 49.05–49.26° E). Montagne d'Ambre reaches an elevation of 1475 m above sea level, is mostly covered by medium elevation moist evergreen forest but also hosts other vegetation types, and is surrounded by drier habitat which constitutes a separation from the other rainforest blocks occurring to its south (Gautier et al., 2018). Most of the massif is protected as Montagne d'Ambre National Park (MANP).

The main transect for the bulk of our sampling ran from ~700 m a.s.l. to almost the peak of the mountain at 1475 m a.s.l., spanning a distance of about 12 km. Additionally, we sampled localities in the Forêt d'Ambre parcel to the north of the park starting at elevations of ~470 m a.s.l., and, for the first time, the west slope of the mountain at ca. 770–1290 m a.s.l.; connecting these additional sampling sites to our main transect gives a total transect length of about 17 km, with a gap between the Forêt d'Ambre sampling points and the main transect of about 4 km which could not be sampled due to safety concerns (related to illegal logging inside the park). Reptiles and amphibians were surveyed by day and at night by torchlight along transects, with additional targeted searches for calling frogs, pitfall trapping, and opportunistic searching.

All research methods reported in this paper complied with the guidelines for field research compiled by Herpetological Animal Use and Care Committee (HACC) of the American Society of Ichthyologists and Herpetologists (ASIH), the Herpetologists' League (HL), and the Society for the Study of Amphibians and Reptiles (SSAR) (HACC, 2004), and adhered to the legal requirements of Malagasy authorities. Approval for this study by an Institutional Animal Care and Use Committee (IACUC) was not required by Malagasy law and approval of sampling procedures was instead included in the fieldwork permit issued by the Ministry of Environment, Direction of the System of Protected Areas (Fieldwork permit N°19117‐MEEF/SG/DGF/DSAP/SCB.Re). Permit numbers for export are in the Acknowledgements.

For all members of the target species (complexes), we took samples in the form of buccal swabs or tiny clips of web, or scales, after treating animals locally with a pain relieving, antiseptic and antibacterial Bactine® spray to reduce animal suffering and avoid infection. Samples were preserved in vials with 100% ethanol. Representative specimens were collected for all encountered species. These voucher specimens were sedated and subsequently euthanized by immersion in or injection of MS‐222 solution, in compliance with the guidelines of the American Veterinary Medical Association Panel on Euthanasia (2020). Tissue samples of these specimens were then taken and stored in 100% ethanol, and the specimens subsequently fixed in 90% ethanol and transferred to 70–80% for long‐term storage. Half of the voucher specimens were deposited in the zoological collection of the Mention Zoologie et Biodiversité Animale of the Université d'Antannarivo (UADBA), and the other half in the Zoologische Staatssammlung München (ZSM) in Germany.

### 2.2 DNA barcoding

DNA from tissue samples was extracted using a standard salt extraction protocol (Bruford et al., 1992). For DNA barcoding, we relied on a segment of the mitochondrial 16S rRNA gene which has been used as a standard marker for molecular identification in Malagasy amphibian and reptiles (e.g. Vieites et al., 2009). For the majority of samples (*N* = 2631), we amplified a short segment of this gene (about 250 bp of a hypervariable stretch) using an amplicon sequencing approach on an Illumina MiSeq instrument with a v2 250 cycle reagent kit, following primers and protocols as in Vences et al. (2016). Raw Illumina reads were demultiplexed and quality‐filtered using the software Quantitative Insights Into Microbial Ecology (QIIME) (Caporaso et al., 2010), and subsequently clustered into sub‐operational taxonomic units (sOTUs) using the deblur workflow (Amir et al., 2017). This workflow ensures that 16S haplotypes differing by single or few nucleotide substitutions are also separated into distinct consensus sequences. For selected probes of each sample and lineage, we also used primers 16Sar‐L and 16Sbr‐H (Palumbi et al., 1991) to amplify and Sanger‐sequence a ca. 550 bp segment that includes the shorter DNA metabarcoding segment plus adjacent stretches. In order to exclude phenomena of mitochondrial introgression into entirely different species, we also sequenced segments of nuclear‐encoded protein‐coding genes for some taxa, in particular the recombination‐activating gene 1 (RAG1) for the focal frog species (see Rasolonjatovo et al., 2020 for primers and protocols) and the oocyte maturation factor Mos (CMOS) for the two chameleon target species using primers CO8 (5′‐GCTTGGTGTTCAATAGACTGG‐3′) and CO9 (5′‐TTGGGAGCATCCAAAGTCTC‐3′) (Han et al., 2004). Sequences obtained via Sanger sequencing were quality‐controlled and edited when necessary, using CodonCode Aligner v. 3.5.6 (CodonCode Corporation).

### 2.3 Generating high‐resolution climate data

To incorporate the role of 90 m‐scale microtopography variation, as well as other high‐resolution geomorphological landscape measurements (i.e. terrain forms: ridge, peaks, valleys etc.) into spatial analyses, we implement a recently developed method to downscale 1 km climate data to 90 m resolution (JLB in preparation). This method is implemented in an open‐source R package entitled *MACHISPLIN* and is freely available at https://jasonleebrown.github.io/machisplin/.

In brief, we used the current 19 bioclimatic variables at 30 arc‐second resolution from Chelsa (Karger et al., 2017). A 90 m resolution digital elevation model (DEM) was obtained from the USGS EarthExplorer database (Farr et al., 2007) and was trimmed to the extent of the study (11.935° S, 48.699° E; 13.163° S, 50.038° E). The trimmed 90 m DEM was used to generate six geomorphological landscape measurements: aspect (the downslope direction), slope (the steepness of the altitude), valley depth (difference between the bottom of the valley and ridge level), channel network distance (distance to nearest channel = stream), topographic wetness index (topographic control on hydrological processes), and geomorphons (common terrain forms). Aspect and slope were generated in ArcMap v10.7 (ESRI). Valley depth, channel network distance, and topographic wetness index were generated in SAGA v8.1.3 (Conrad et al., 2015). Geomorphons were created in Grass GIS v8.0 (GRASS Development Team, 2022) using a cell resolution of 30 m. The 90 m DEM and five 90 m geomorphological landscape measurements were then used as high‐resolution covariates in *MACHISPLIN* to downscale all 30 arc‐second bioclimatic variables to 90 m using the default parameters.

### 2.4 Community similarity

**FIGURE 1:**
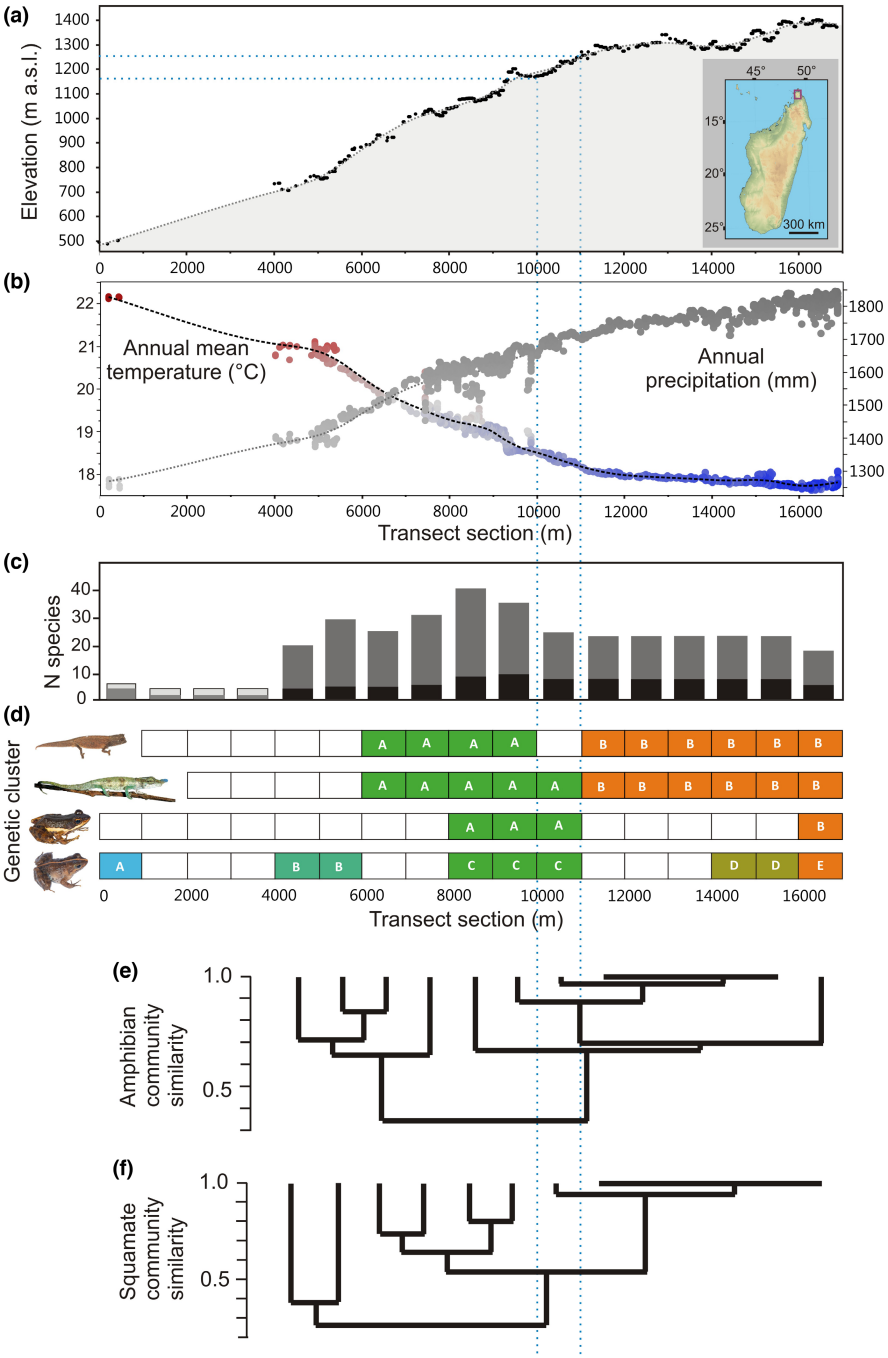
Characteristics of the transect sampled at Montagne d'Ambre. (a) Elevation in m above sea level vs. linear transect distance in meters. The inset map shows the island of Madagascar, with the position of Montagne d'Ambre in the far north marked by a purple rectangle. (b) Annual precipitation and annual mean temperature for all sampling points along the transect, extracted from high‐resolution bioclimatic data. (c) Species richness (number of species) inferred for each 1000 m section of the transect. Numbers refer to transect sections that were regularly sampled and were included in statistical analyses; the first four sections were only incompletely sampled. The darker portions of the bars represent species endemic to the Montagne d'Ambre Massif. (d) Simplified representation of genetic clusters of the target species along the transect, based mostly on microsatellites but also mitochondrial DNA; see Figure 2 for details. Colors of genetic clusters roughly agree with those used in the STRUCTURE plots in Figure 2. (E, F) Dendrograms calculated from Sørensen (Bray‐Curtis) similarities using species presence/absence data of amphibian and reptile communities along the transect. The two horizontal and vertical blue lines mark the transect section and elevations where all of the target species show a break either in genetic clusters or occurrence, and a major drop in species diversity toward higher elevation is observed.

For analysis of community similarity, we subdivided the sampling transect into sections of 1 km linear distance, which encompass substantial variation in elevation and climatic conditions (Figure 1a,b). For each transect section, we summarized the presence or absence of all species, based on GPS data of recorded specimens and their field and DNA barcode identification. To decide which species was present in which section we took the first and last occurrence of each species and assumed its presence in between those two points. This procedure assumes homogeneity of distribution of the species which was not ascertained in every case, but was necessary to account for rareness, that is, species for which only few records were available.

We then used a Mantel test to analyze whether differences in assemblage composition among transect sections were related to differences in bioclimatic variables. Similarity matrices were calculated based on Sørensen (Bray‐Curtis) similarities using species presence/absence data for compositional data and the Euclidean distance for differences in bioclimatic data (Faith et al., 1987; Magurran, 2004). Mantel tests were performed based on Pearson's product–moment correlation. *p*‐values were obtained from 9999 permutations. Dendrograms were derived from the similarity matrices using the unweighted pair‐group clustering method (UPGMA) (Sokal & Michener, 1958) and Sørensen (Bray‐Curtis) distances to visualize differences in species composition (and assemblage turnover) between assemblages among different transect sections. These statistical analyses were performed in PAST 3.26 (Hammer et al., 2001).

### 2.5 Microsatellite library development

We extracted genomic DNA from muscle tissue samples from four specimens of the main target species: two frogs (*Mantidactylus ambreensis, M. bellyi*) and two chameleons (*Brookesia tuberculata, Calumma linotum*). The pooled DNA per species was then submitted to the Sequencing Genotyping Facility, Cornell Life Sciences Core Laboratory Center (CLC), USA, for the development of microsatellite libraries (see Appendix S1 for details of wet lab methods).

SeqMan NGen version 11 (DNASTAR) was used for raw read assembly, and the program msatcommander 1.0.8_beta (for macOS) (Faircloth, 2008) was employed to scan the assembly for microsatellite loci and to automatically design primer pairs. After removal of duplicates, the constructed libraries contained the following numbers of proposed microsatellite markers with minimum consecutive perfect repeat lengths of at least six (12 bp) for any dimer and at least five for any trimer, tetramer, pentamer or hexamer, PCR product size of 150–450 bp, and duplicates removed: 11,217 markers for *B. tuberculata*, 7954 for *C. linotum*, 3707 for *M. ambreensis*, and 14,545 for *M. bellyi*. The full libraries are available as Appendix S1 (Tables S4–S6) and from Figshare under DOI 10.6084/m9.figshare.14618175.

Of these libraries, we chose for each species 20–30 loci based on the following criteria (Perl et al., 2018): (i) tetrameric, (ii) repeat motif between 10 and 15, (iii) less than 1000 reads, as deep coverage could indicate multiple copies, and (iv) GC content of 50 (Table S1). We tested these loci for successful amplification and for yielding unambiguously scorable and polymorphic PCR products. After exclusion of markers that did not or irregularly amplify or for which null alleles were detected, we selected 13 microsatellite markers for *B. tuberculata*, 8 for *C. linotum*, 10 for *M. ambreensis*, and 7 for *M. bellyi* (Table S1).

### 2.6 Microsatellite genotyping

Microsatellites were amplified following the nested protocol of Schuelke (2000) with modifications, and fragment analysis was performed on an ABI 3130xl Genetic Analyzer (see Appendix S1 for details). We called alleles with GeneMarker® (SoftGenetics, State College, PA, USA); ambiguous calls were either excluded if poor quality, or rounded up to the next unambiguous allele size. We tested for Hardy–Weinberg equilibrium, linkage disequilibrium, and F_ST_ values between locality groups in Arlequin (Excoffier et al., 2005) under Bonferroni correction. Linkage disequilibrium was detected in all locality groups of all species, but in general, the linked loci differed from each other. Only in *B. tuberculata* and *C. linotum* did we observe four and one pair of loci, respectively, with linkage disequilibrium across all locality groups, confirming that with these exceptions, most or all loci analyzed can be considered to be unlinked in the genome. We used MICRO‐CHECKER version 2.2.3 (van Oosterhout et al., 2004) to test for potential scoring errors, large allele dropout, and the presence of null alleles. Scoring errors or large allele dropouts were not observed, but potential null alleles due to an excess of homozygotes could not be excluded for most loci of all target species. Full lists of newly characterized microsatellite libraries as well as allele files used as STRUCTURE input have been deposited in the Figshare repository under DOI 10.6084/m9.figshare.14618175.

### 2.7 Population genetic analysis

We analyzed population structure with the software STRUCTURE version 2.3.4 (Pritchard et al., 2000) under the assumption of an admixture model with correlated allele frequencies and LOCPRIOR. We compared the number of clusters (K) with 500,000 Markov Chain Monte Carlo (MCMC) iterations and a burn‐in of 50,000, repeating each assessment of K ten times. To assess the optimal number of clusters, we applied the ΔK method by Evanno et al. (2005) using STRUCTURE HARVESTER (Earl & Von Holdt, 2012) as an initial guideline. However, since our goal was to assess if any kind of genetic differentiation, even if very weak, across space and elevation exists, we subsequently manually inspected STRUCTURE plots with K values close to those suggested by the ΔK comparison, and chose those that suggested the clearest pattern of geographical clusters. Analyses with the preferred K value (typically those with the highest likelihood) were repeated using the CLUMPP procedure of Jakobsson and Rosenberg (2007) to test for consistency between approaches.

Principal component analysis (PCA) of microsatellite allele data was carried out with the packages *diveRsity* v. 1.9.90 (Keenan et al., 2013) and *adegenet* v. 2.1.5 (Jombart, 2008) in the R environment (R Core Team, 2020), following Jombart et al. (2009).

Our goal was to maximize possible weak signals of population differentiation in the microsatellite data to understand at which elevation potential genetic differences occur—including very weak ones—rather than obtaining a clustering without a‐priori assumptions. We therefore used locpriors in our final STRUCTURE runs, determined as follows: For *B. tuberculata*, we defined three a‐priori populations, corresponding to sampling sites separated by an obvious gap (mostly due to unequal sampling effort) and to the population size determined by the Evanno method, plus samples from the west slope as an additional population; in *C. linotum*, we also defined three a priori populations based on sites dominated by each of the three main mitochondrial haplotypes; for *M. ambreensis/M. ambony*, and *M. bellyi*, we defined four and six a‐priori populations based on an obvious geographical clustering of collection sites. In addition, we also performed the same analyses for all taxa without setting locpriors to understand if the inferred clusters would also be recovered without a‐priori assumptions.

Besides the microsatellite analysis, we also examined differentiation of target species populations in mitochondrial alleles. Sequences were aligned and trimmed in MEGA7 (Kumar et al., 2016), and relationships among haplotypes visualized as networks. For this, we reconstructed maximum likelihood trees in MEGA7 (Kumar et al., 2016), and these trees were then used together with the respective alignments as input for Haploviewer (written by G. B. Ewing; http://www.cibiv.at/~greg/haploviewer), a software that implements the methodological approach of Salzburger et al. (2011).

### 2.8 Morphometric analyses

To understand possible morphological adaptation of herpetofauna related to elevation at Montagne d'Ambre, a series of morphometric measurements were taken from the same individuals studied genetically, including the frog species (*M. ambreensis/M. ambony, M. bellyi*) and one of the chameleon species (*Calumma linotum*). Furthermore, morphometric data were also taken from two additional chameleon species (*Calumma amber, C. ambreense*) occurring over a relatively wide elevational range of our transect. Due to a lack of time and resources in the field, no morphometric data of *B. tuberculata* could be taken. All measurements were taken on live animals. Measurements taken for the frogs were snout–vent length (SVL), head length, maximum head width, horizontal eye diameter, tympanum diameter, humerus length, forearm length, thigh length, tibia length, tarsus length, and length of the third toe. For the chameleons, we measured snout–vent length, tail length, head length from snout tip, thigh length, hindlimb length, chest length, length of rostral appendage, snout–casque distance (from snout tip to posterior edge of casque), and casque height.

Here, we only present a selected number of morphometric comparisons to highlight the existence of elevational differentiation, especially in body size, across the various species. For *M. ambreensis/M. ambony*, and *M. ambreensis*, only a small number of specimens were measured from a limited elevational range, and these species are thus not further considered. For *M. bellyi*, a detailed morphometric comparison of individuals belonging to different clusters has been published by Rasolonjatovo et al. (2022) and is therefore not repeated here. For *Calumma amber* and *C. ambreense*, only a simple correlation of body size with elevation was performed since no information on genetic clusters was obtained for these two species. Furthermore, we limited our primary body‐size‐related analyses to adult males since maturity in the male sex is easier to ascertain in both mantelline frogs (by the presence of femoral glands) and chameleons (by an enlarged tail base due to the presence of hemipenes, and by the presence of cephalic ornaments) than in females (Glaw & Vences, 2007), where we cannot exclude that subadult females or even males were scored as females, given the restraints imposed by nocturnal examination in the field.

Graphs and simple correlation analyses of morphometric values and elevation were computed in JMP 13.0 (SAS Institute Inc.). PCAs on the SVL and size‐residuals of other measurements of *C. linotum* were performed using the prcomp() function from the stats package in R version 4.1.3 (R Core Team, 2020), with the data scaled and centered.

### 2.9 Niche divergence tests

We performed niche divergence tests using the R package *humboldt* (Brown & Carnaval, 2019). A significant niche divergence test concludes that the two occupied niches are statistically different and likely the result of niche divergence. For this test, we characterized the climate and topography of the research area by sampling 30,000 points from the 90 m climate data from an area north of −12.65 degrees latitude.

### 2.10 Concordance between community and species differentiation

We tested the hypothesis of differences in species composition between elevational sections of the transect by performing permutational multivariate analyses of variance (perMANOVA) (Anderson, 2001; McArdle and Anderson, 2001) in PAST 3.26 (Hammer et al., 2001). The grouping of transect sections was based on the genetic clusters found for *Calumma linotum* (group 1 consisting of transect sections between 6001 and 11,000 m [ca 900–1250 m a.s.l.], group 2 consisting of transect sections between 11,001 and 16,000 m [ca 1250–1350 m a.sl.], and group 3 consisting of the transect section between 16,001 and 17,000 m [ca 1300–1350 m a.s.l.]) and for *Brookesia tuberculata*, respectively (group 1 consisting of transect sections between 6001 and 10,000 m [ca 900–1200 m a.sl.], group 2 consisting of transect sections between 11,001 and 17,000 m [ca 1250–1350 m a.s.l.]). In *M. bellyi* and *M. ambreensis/M. ambony*, the genetic clusters were restricted to only a few (i.e. one to three) transect sections. Therefore, we did not apply perMANOVA for testing for differences between genetic clusters due to the low number of statistical replicates (i.e. transect sections).

This non‐parametric permutation‐based variant of MANOVA partitions sums of squares of multivariate data equivalent to univariate ANOVA and the pseudo F statistic can be calculated directly from any distance measure (Anderson, 2001). We performed perMANOVA based on Sørensen (Bray‐Curtis) similarities using species presence/absence data. *p*‐values were obtained from 9999 permutations.

## 3 RESULTS

### 3.1 Community turnover

Our herpetological inventory of Montagne d'Ambre yielded 2631 records (1241 verified by DNA barcoding) corresponding to 34 species of amphibians and 48 species of reptiles, spanning an elevational range of ca. 470–1470 m a.s.l. We made first records on Montagne d'Ambre for the frog *Boophis albilabris* and the snake *Micropisthodon ochraceus*, which constitute major range extensions for both species, as well as a new population of *Stumpffia megsoni*, and the new species *S. bishopi* (Rakotoarison et al., 2022) and *Lygodactylus tantsaha* (Vences et al., 2022). Many of the species recorded were restricted to particular elevations, leading to substantial differences both in community composition and in species richness along the sampling transect. Not considering the first 4 km of the transect, where only limited sampling was possible, species richness of amphibians and reptiles doubled from 21 species at the first transect section (~4000–5000 m along the transect, ca. 700 m a.s.l., Figure 1a,c) to its peak with 41 species at the fifth transect section (~8000–9000 m along the transect, about 1000 m a.s.l.), and then again decreased above 1100 m elevation (beyond 9000 m along the transect) to about 20 species (Figure 1c). Counting only species that according to current taxonomy are endemic to the Montagne d'Ambre Massif, a similar trend was observed, with a peak of 9–10 species at the fifth and sixth transect section. Again not considering the incompletely sampled first 4 km of the transect, the percentage of local endemics in the community increased with elevation, from 19% of the total species numbers in the first section to 33–35% in the sections 7–13, corresponding to elevations >1100 m.

We found a significant correlation between bioclimatic variables and compositional differences of amphibian and reptile assemblages combined (Mantel‐test: *r* = .88, *p* < .0001) and of reptiles only (*r* = .88, *p* < .0001). A dendrogram of amphibian community similarity (Figure 1e) suggested a major turnover between communities at lower elevations (transect sections 1–4, corresponding to ca. 700–1000 m a.s.l.) and higher elevations (transect sections 5–13, >1000 m a.s.l.). For reptile communities (Figure 1f), the main turnover separated transect sections 1–2 (ca. 700–800 m a.s.l.) from sections 3–13 (>800 m a.s.l.), with a secondary break between sections 3–6 and 7–13, corresponding to ca. 800–1150 m a.s.l. vs. >1150 m a.s.l. (see also Figure S1). Thus, there is strong evidence for herpetofaunal community turnover on Montagne d'Ambre related to elevation, suggesting that this ecotone is significant.

### 3.2 Gene flow of Montagne d'Ambre endemics

For all four target species or species complexes (*Brookesia tuberculata, Calumma linotum, Mantidactylus bellyi*, and *M. ambreensis/M. ambony*), we obtained evidence for genetic differentiation along the elevational transect sampled at Montagne d'Ambre, but the depth of divergence and support for it was strongly different among the species.

**FIGURE 2:**
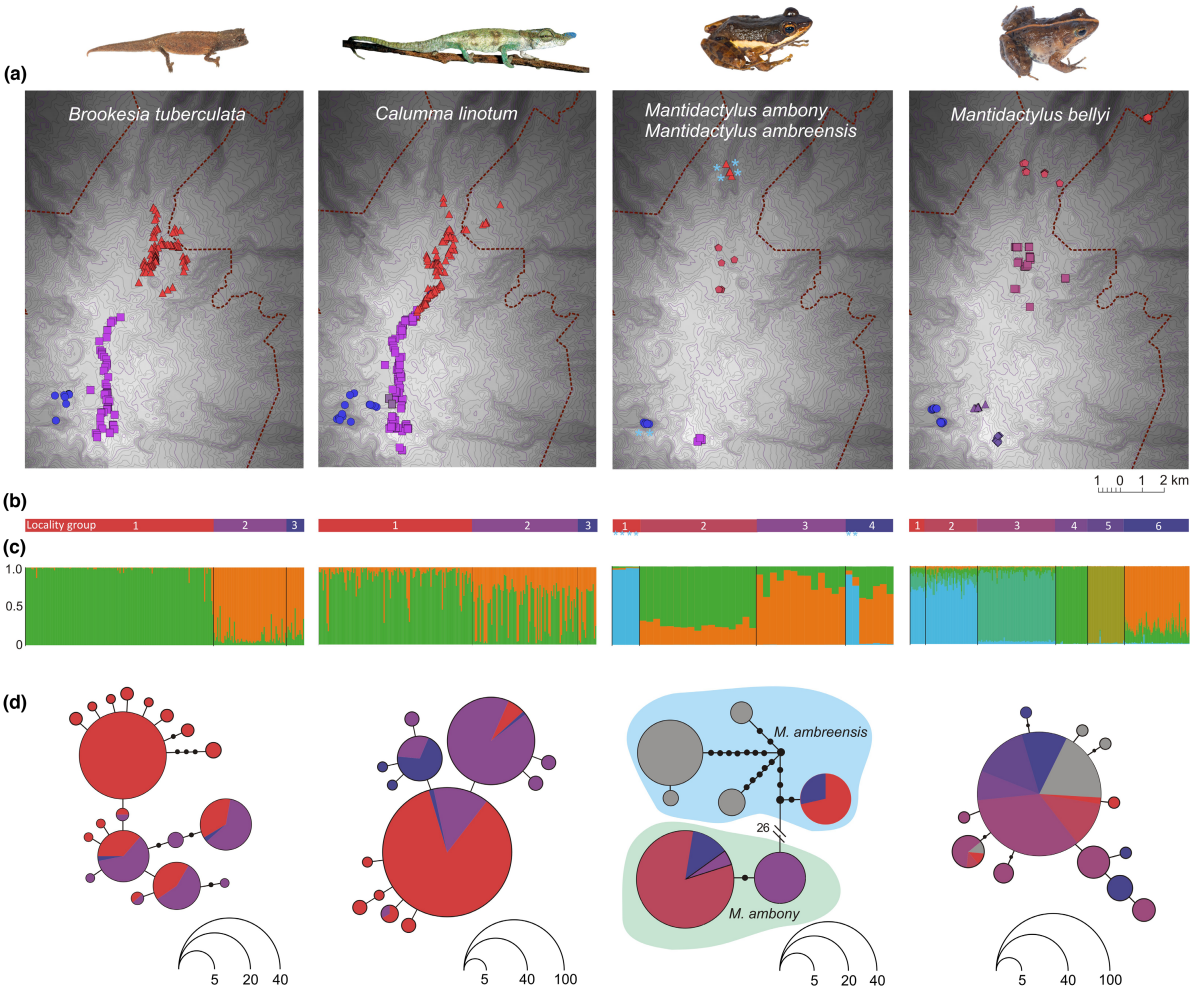
Genetic differentiation in the four target species (complexes) of amphibians and reptiles on Montagne d'Ambre. Panels arranged in each column refer to the same species or species complex. (a) Maps showing sampling points for each species along the transect. Colors correspond to (b) a‐priori locality groups that were defined based on obvious geographic clustering of sampling, in the case of *C. linotum* combined with preliminary analysis of mitochondrial haplotypes. (c) STRUCTURE plots showing assignment of individuals to genetic clusters, each bar corresponds to one individual, and these belong to geographic groups as shown in (b). (d) Haplotype networks reconstructed from segments of the mitochondrial 16S rRNA gene, colors corresponding to individuals of the respective geographic groups and gray color denoting samples of imprecise localities or from sites not in Montagne d'Ambre. Note that for the species complex *M. ambreensis/M. ambony*, light blue color in (c) and (d) denotes samples of *M. ambreensis*, and other colors denote *M. ambony*.

For *B. tuberculata*, the analysis of 13 microsatellite markers for 234 samples revealed a relatively clear signal of two genetic clusters as for higher numbers of clusters, the likelihood variation among replicate runs of STRUCTURE increased (Figure S2) and ΔK became distinctly lower (Figure S3). The STRUCTURE runs without locprior revealed the same two clusters but with a less clear‐cut signal (Figure S4). The two genetic clusters on the east slope corresponded to samples from elevations between 887 and 1170 m a.s.l. (a‐priori locality group 1) vs. 1260–1455 m a.s.l. (locality group 2), separated by a gap in which no specimens of these miniaturized ground chameleons were sampled; samples from the western slope 956–1224 m a.s.l. (locality group 3) were included in the second genetic cluster. All but two samples from the lower elevation cluster were assigned with high probability values, whereas numerous samples of the higher elevation cluster had somewhat lower assignment probabilities (Figure 2 and Figure S5). The PCA (Figure S9) confirmed a quite distinct separation between the two genetic clusters. STRUCTURE runs with higher K values did not reveal additional geographical clusters (Figure S4). The lower elevation cluster contained a large variation of mitochondrial haplotypes, whereas the higher elevation cluster was only represented by one haplogroup (Figure 2).

For the larger sized, arboreal chameleon *C. linotum*, only very weak indications of nuclear genetic structure were found. For 219 samples collected along almost the entire transect without any major sampling gap, analyzed for eight microsatellite markers, the value of ΔK decreased after K = 3. However, likelihood variation among replicate runs was high for K = 2, 3, 4 and 5 (Figures S2 and S3). Inspection of STRUCTURE plots revealed a weakly expressed trend of geographic clustering for K = 2, but no such trend for higher K values (Figure S4). The two‐cluster signal disappeared when running STRUCTURE without locprior, confirming the weakness of this trend (Figure S4). The PCA plot (Figure S10) also confirmed that the difference between the genetic clusters is weak, with a substantial overlap of the respective samples. The two genetic clusters for K = 2 on the eastern slope corresponded to samples from 811–1270 m a.s.l. (corresponding to locality group 1) vs. samples from 1268–1482 m (locality group 2); samples from the western slope (914–1283 m a.s.l. locality group 3) were included in the second genetic cluster. Many samples, however, had an equal or higher assignment probability to the other cluster; this applied to ca. 8 samples from the lower elevation cluster, and over 30 samples in the higher elevation cluster (Figure 2 and Figure S6). From a mitochondrial perspective, the lower elevation cluster was dominated by one main mitochondrial haplotype and a few singleton haplotypes, whereas the higher elevation cluster was mainly represented by a different haplogroup, but some haplotype sharing between the two clusters occurred (Figure 2).

For the *M. ambreensis/M. ambony* complex, only a relatively low number of 41 samples was genotyped for 10 microsatellites. Of these, six samples corresponded to *M. ambreensis*, and 35 to *M. ambony*. The two species which are genetically highly distinct for both mitochondrial and genomic datasets (Rasolonjatovo et al., 2020) were also neatly separated by the microsatellite data (Figure 2 and Figure S7). Likelihood values were highly variable among replicate runs for all values of K > 1 (Figure S2). Inspection of ΔK values revealed a disparate pattern where the highest value corresponded to an unrealistically high K = 7 (Figure S3), but a clear geographical/taxonomic clustering pattern was only revealed for K = 2 (separating *M. ambony* from *M. ambreensis*) and K = 3 (additionally separating weakly *M. ambony* into two geographic clusters, occurring at 1036–1196 vs. 1133–1375 m a.s.l. on the eastern slope, plus 941–966 m a.s.l. on the western slope) (Figure S4). The STRUCTURE runs without locprior confirmed the separation among the two species, but did not reproduce the weak clustering pattern within *M. ambony* (Figure S4). The PCA (Figure S11) also confirmed the expected clear divergence between *M. ambony* and *M. ambreensis*, whereas the divergences between the genetic clusters of *M. ambony* are only weakly recognizable. Mitochondrial DNA did not provide a clear separation between the two genetic clusters, although the higher elevation cluster (but not specimens from the western slope) was dominated by one haplotype not found in the lower elevation cluster (Figure 2).

For *M. bellyi*, analysis of 236 samples for seven microsatellite markers revealed clear evidence for geographic structure. This stream‐breeding species did not occur continuously along our transect, but was bound to locations with streams, which were geographically clustered. While ΔK was highest for K = 3, likelihood values increased and were stable among replicate runs until K = 5 (Figures S2 and S3), and STRUCTURE plots suggested clear geographically separated clusters until K = 5; for higher K values, the pattern became less clear. The genetic clusters for K = 5 fully corresponded to the a‐priori defined geographical clusters, except for samples of geographical groups 1 and 2 which formed one genetic cluster (and were also not separated with higher K values) (Figure S4). Genetic clusters were distributed at 467–774 m a.s.l. (genetic cluster A, corresponding to locality groups 1 and 2), 997–1192 m a.s.l. (genetic cluster B, corresponding to locality group 3), 1244–1269 m a.s.l. (genetic cluster C, corresponding to locality group 4), 1277–1394 m a.s.l. (genetic cluster D, corresponding to locality group 5), and 923–980 m a.s.l. (genetic cluster E, corresponding to locality group 6 on the western slope). The STRUCTURE runs without locprior revealed a very similar clustering as with locprior (Figure S4). PCA confirmed that there are three highly distinct genetic groups, where clusters C and D (locality groups 4 and 5) each are separate from each other and from a main group containing the other clusters (Figure S12). Assignment probabilities were relatively high in most cases, and no sample was assigned to another geographical cluster with a probability >50%. Mitochondrial DNA did not provide any indication of separation among the microsatellite‐based genetic clusters, with one main haplotype shared among all clusters (and also prevalent in populations outside of Montagne d'Ambre), and a few other rare haplotypes and singletons restricted to certain clusters (Figure 2).

### 3.3 Morphological and ecological niche differentiation across populations

Niche divergence tests detected significant differences (*p* < .01) in the bioclimatic envelopes among all genetic clusters and a‐priori geographical locality groups of the four target species *B. tuberculata, C. linotum, M. ambony*, and *M. bellyi*, except for the geographical groups 4 and 5 of *M. bellyi* which had similar bioclimatic characteristics (*p* = .54). All background tests were significant among all comparisons and support the idea that there is sufficient power for niche divergence tests to detect environmental differences among comparisons, including the geographical groups 4 and 5 of *M. bellyi*.

**FIGURE 3:**
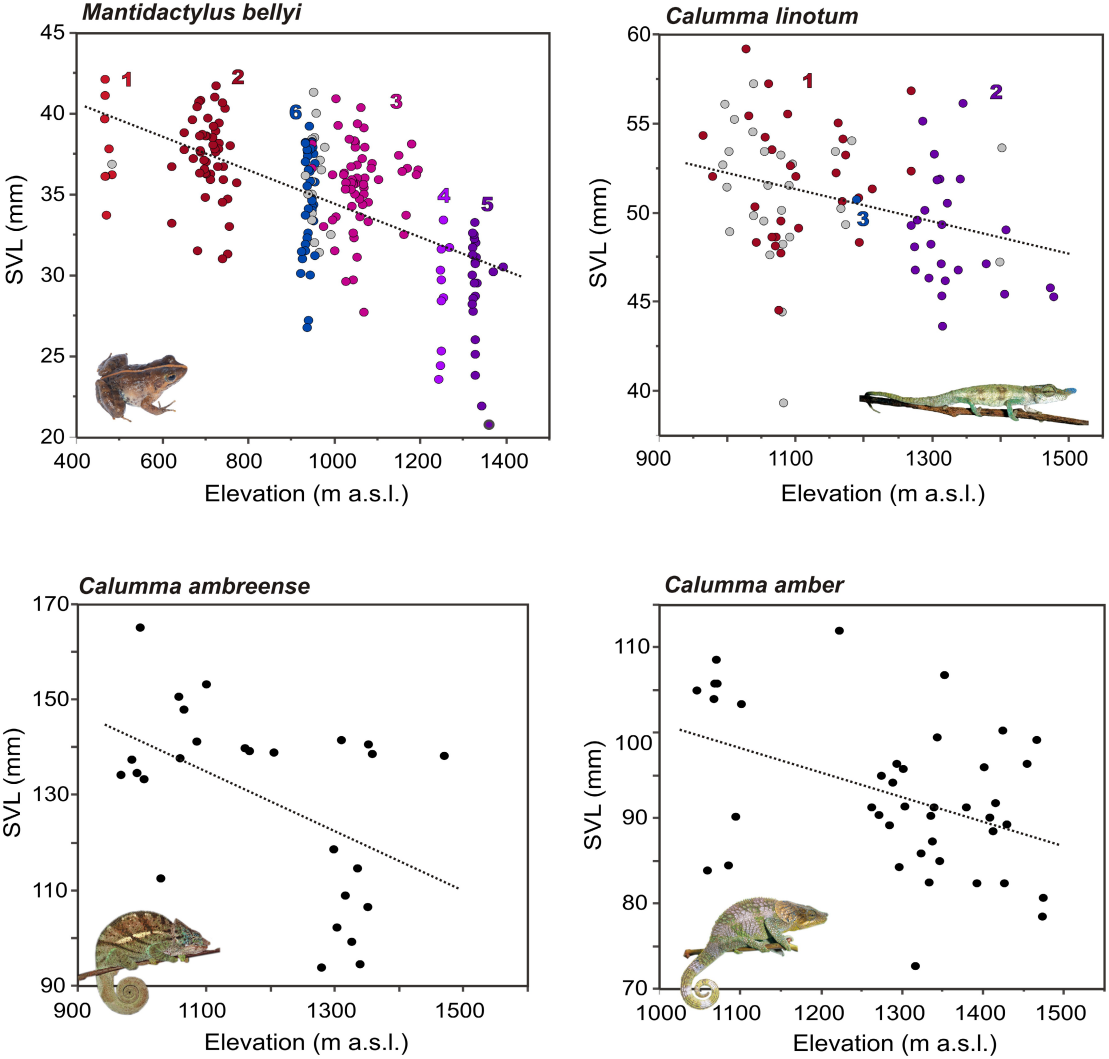
Body size of four selected species of amphibians and reptiles occurring over a wide elevational range on Montagne d'Ambre relative to elevation. All measurements are from adult males and were taken from November 2017 to January 2018. In the plots of *M. bellyi* and *C. linotum*, dots are colored according to locality groups (1–6 for *M. bellyi*, 1–3 in *C. linotum*, as in Figure 2); gray dots are unassigned individuals that were not genotyped.

Several species showed trends of morphometric differentiation within the study area. Shape differences between genetic clusters were analyzed for *M. bellyi* (by Rasolonjatovo et al., 2022; for detailed results see there) and showed a weak separation in multidimensional space for specimens from locality groups 4 and 5 (genetic clusters C and D) relative to all other locality groups, in separate analyses for both males and females. For *C. linotum*, a PCA on all size‐corrected morphometric measurements showed that specimens from genetic cluster B (high elevation) clustered more tightly while those from genetic cluster A (lower elevation) had a higher variation; however, individuals from both genetic clusters largely overlapped in morphometric multivariate space (Figure S14). While morphometric differences among genetic clusters in these two species were weak (*M. bellyi*) or basically absent (*C. linotum*), we detected a consistent statistically significant effect of elevation on body size in one frog and three chameleon species (Figure 3): *M. bellyi*, (*N* = 207, correlation coefficient *R* = −.60, *p* < .0001), *C. amber* (*N* = 41, *R* = −.43, *p* = .0057), *C. ambreense* (*N* = 23, *R* = −.49, *p* = .0106), *C. linotum* (*N* = 83, *R* = −.33, *p* = .0021). In *C. linotum*, a possible inverse trend of smaller body size at lower elevations was observed in females (Figure S15), which, however, could also be due to subadults incorrectly scored as females.

### 3.4 Alignment of genetic turnover with ecological and community turnover

While the samples of our *Mantidactylus* target species were rather patchily distributed, sampling of the two chameleons was more complete along the transect, except for a sampling gap in *B. tuberculata*. To test for a correlation between overall community patterns and genetic clusters in the target species, we subdivided the transect into two sections, respectively, corresponding to the genetic clusters in *B. tuberculata* and *C. linotum*. For transect sections defined by genetic clusters of *C. linotum*, differences in community composition were significant for the combined assemblages of amphibians and reptiles (perMANOVA, *F* = 3.71, *p* = .016), and for reptile assemblages alone (perMANOVA, *F* = 5.07, *p* = .008). For transect sections defined by genetic clusters of *B. tuberculata*, community composition significantly differed for the combined assemblages of amphibians and reptiles (perMANOVA, *F* = 12.15, *p* = .005), and for reptile assemblages alone (perMANOVA, *F* = 22.03, *p* = .005). Also, an NMDS analysis revealed distinct dissimilarity patterns in species composition between transect sections defined by genetic clusters in the two chameleons (NMDS – plot not shown). The stress values (*C. linotum*: 0.09 for combined amphibian and reptile assemblages, 0.15 for only reptile assemblages; *B. tuberculata*: 0.12 for combined amphibian and reptile assemblages, 0.20 for only reptile assemblages) of the final two‐dimensional solution indicate a reasonable preservation of ordering relationships of the multidimensional among‐transect dissimilarities.

## 4 DISCUSSION

Madagascar has served as model to develop numerous hypotheses of diversification mechanisms (e.g. Colwell & Lees, 2000; Craul et al., 2007; Dewar & Richard, 2007; Goodman & Ganzhorn, 2004; Kuhn et al., 2022; Lees et al., 1999; Raxworthy & Nussbaum, 1995; Wilmé et al., 2006; Wollenberg Valero, 2015), as partially summarized in Vences et al. (2009) and Brown et al. (2014). Most of these studies were based on either patterns of species distributions, phylogenies, or both. Also in other tropical regions and globally, much work has concentrated on combining phylogenetic and geographic patterns, for example, to identify montane areas as species pumps (Hutter et al., 2013; Toussaint et al., 2013) or to assess speciation rates across the global latitudinal diversity gradient (Igea & Tanentzap, 2020; Mittelbach et al., 2007; Wiens et al., 2006). Research with a full or partial focus on ecological speciation, especially in tropical systems, often relies mainly on phylogenetic or phylogeographic analyses (Beheregaray et al., 2015). Studies examining putative ecological speciation in natural replicates typically targeted systems with repeated divergences of a taxon over several similar ecotones or environmental gradients (e.g. Rosenblum & Harmon, 2011). Here, we have instead looked at the influence of a common environmental gradient on a variety of co‐occurring taxa (reptiles and amphibians). Amphibians and reptiles, due to their often‐high population densities, especially in Madagascar, are relatively easy to sample and therefore offer an opportunity to comparatively study population structure and turnover across landscape gradients at a scale of meters, especially if they are continuously distributed in the forest, as in the two target chameleon species in our study.

### 4.1 Community turnover across the ecotone — A common pattern in Madagascar

A recurrent finding from inventory work across elevational gradients in Madagascar is that species richness of multiple organism groups peaks at mid‐elevations (e.g. birds, Goodman et al., 2000; tenrecs, Goodman & Jenkins, 2000). Averaged over the entire herpetofauna of Madagascar, Brown et al. (2016) found a peak in amphibian richness at around 1000 m a.s.l., whereas in reptiles, species numbers peaked in lowland areas due to the large number of species occurring <500 m a.s.l. in western Madagascar. Fine‐scale data from Montagne d'Ambre (Figure 1) provide an almost perfect match to these previous observations and demonstrate, for this mountain's herpetofauna, a richness peak between 1000 and 1100 m a.s.l., and an increase of the proportion of local endemics in the community above this elevation. Furthermore, at this same elevation, we also observed a main point of turnover among herpetofaunal communities, especially for amphibians, whereas in squamates, the main turnover took place at ca. 800 m a.s.l. The observed community turnover at elevations of roughly 800–1000 m a.s.l. at Montagne d'Ambre appears to coincide with categorical herpetofaunal turnover boundaries calculated by Brown et al. (2014, 2016) using generalized dissimilarity modeling at larger spatial scales in Madagascar. These recurrent discontinuities between low‐ and mid‐elevation herpetofaunas suggest that the richness peak at ca. 1000 m a.s.l. may not be a simple, fully stochastic mid‐domain effect where the maximum range overlap of a randomly distributed community of species is found in the center of a domain (in this case at a two‐dimensional center, i.e. mid‐elevation) (Colwell & Lees, 2000; Lees et al., 1999). Instead, it appears that there are two rather well‐defined assemblages of species, one specialized to lower, one to higher elevations, and the richness peak at ca. 1000 m a.s.l. is caused by the range overlap of the species belonging to either of these two assemblages.

### 4.2 Parallel but unequal differentiation of multiple taxa across the ecotone

All of our focal taxa showed some degree of genetic and morphological divergence over the Montagne d'Ambre elevational gradient. The continuously distributed species studied, the two chameleons *Brookesia tuberculata* and *Calumma linotum*, were divided each into two genetic clusters, although the evidence for the larger sized (and thus putatively more mobile) *C. linotum* was weak. In *B. tuberculata*, the two clusters were divided by a sampling gap that may partly represent a true discontinuity in the distribution of this species. The separation between the two clusters in both chameleon species was found at around 1200 m a.s.l. In the two frogs, *Mantidactylus bellyi* and *M. ambony*, populations above 1200 m a.s.l. also belonged to genetic clusters differing from those at lower elevations. Although this separation is at slightly higher elevation than the community richness peak and the main turnover point, community composition was different in transect sections defined by the genetic clusters of the two chameleon target species, providing some indication that the factors underlying community divergence are also involved in the origin and maintenance of the genetic breaks observed.

While we found at least some weak evidence for genetic structure of our four target species (complexes) distributed over relatively wide elevational ranges (>900 m in the case of *M. bellyi*), the extent of structuring differed among species. The frog *M. bellyi* had clear differences among most locality groups, whereas the chameleon *C. linotum* only showed a weak, poorly detectable differentiation (if any) between two clusters. These differences might be explained by a combination of factors. In the case of *M. bellyi*, its habitat (streams) is not continuously available along the transect sampled, and the stream‐less forest in between locality groups could therefore act as a barrier for gene flow in this semi‐aquatic frog species (Rasolonjatovo et al., 2022). In the two chameleon species, the larger and more continuously distributed species (*C. linotum*) had a less clearly detectable structure than the miniaturized species (*B. tuberculata*). It is well known that small‐sized species of amphibians and reptiles have on average a lower dispersal ability, as indicated by smaller ranges (Brown et al., 2016) as well as slower dispersal rates (Li & Wiens, 2022) and stronger phylogeographic structure (Hancock & Hedrick, 2018; Pabijan et al., 2012; Rodríguez et al., 2015). Small body size might sometimes also lead to a higher diversification rate (Wollenberg et al., 2011). The intrinsic body size factor could underlie the different strength of genetic clustering in *C. linotum* vs. *B. tuberculata*. Additionally, the more arboreal habits of *C. linotum* may facilitate crossing terrestrial barriers via arboreal routes.

Coincident divergence of intraspecific lineages in four different taxa, in parallel with a turnover in overall community composition, across the same ecotone or environmental gradient, provides initial indication for a role of that ecotone in generating and maintaining differentiation. Unfortunately, however, our data do not allow to conclusively demonstrate that genetic breaks of our target taxa are significantly associated with community turnover along the transect, despite this being one of the initial aims of this study, and despite suggestions of a reviewer to statistically test this hypothesis. The small sample size (*N* = 4 target species with population genetic data), weakness of genetic clustering in *C. linotum* and *M. ambony*, and idiosyncratic patterns of sampling gaps across the transect prevent us from statistical testing via, for example, resampling procedures.

Also, our data are not conclusive in identifying the processes underlying this differentiation. Although our analysis revealed the presence of genetic clusters that occupy different elevational bands and differ in their bioclimatic niches, in both chameleon species, the higher elevation clusters reach lower elevation on the west slope of the mountain, well overlapping with the elevational range of the low‐elevation clusters from the northern slope. This leaves the possibility that the differentiation among these is not primarily due to ecological processes, but could instead be due to microallopatry on different parts of the mountain. A microallopatric scenario is particularly likely for the stream‐breeding *M. bellyi*, where the breaks between most genetic clusters are related to areas without suitable habitat. Indeed, genetic clusters of this species were coincident with the differentiation seen in depth and width of the streams they inhabit (Rasolonjatovo et al., 2022). On the other hand, the fact that genetic clusters of *M. bellyi* differ in some morphological and bioacoustic traits (Rasolonjatovo et al., 2022) would rather support that ecological adaptation is involved in their differentiation.

### 4.3 Morphological differences within species across a shared ecotone

The consistent elevational influence on body size of various amphibian and reptile species on Montagne d'Ambre (Figure 3) also suggests that different environmental conditions along the elevational gradient on this mountain influence the biology of these animals. According to Bergmann's rule (Bergmann, 1847), body size of endotherms increases with colder climate, and thus with elevation. In ectotherms, particularly amphibians and reptiles, mixed evidence has been found for this rule, including weak or absent effects in salamanders (Adams & Church, 2008) and amphibians in general (Ashton, 2002), significant effects in turtles (Ashton & Feldman, 2003), and tendencies for a reverse Bergmann's rule in squamates (Ashton & Feldman, 2003). Also in a global comparison at the species level, reverse Bergmann's effects were apparent, but probably driven by assemblage structure (Slavenko et al., 2019). In amphibians, such an inverse relationship, that is, decreasing body size with increasing elevation, has been observed in selected case studies as well, both within clades of related species (Hu et al., 2011) and within species (Hsu et al., 2014) as in our study (Figure 3). One of these works (Hsu et al., 2014) found intersexual differences in this relation, which in their study species was only observed in males, reminiscent of the situation in *C. linotum* at Montagne d'Ambre. However, without being able to reliably distinguish adult females from subadults, we cannot ascertain if the observed trend in *C. linotum* reflects a biological reality. Further in‐depth study will be needed to establish whether the clear trend of decreasing body size with increasing elevation in the males of three chameleon species and one frog species (Figure 3) reflects (i) a true adaptation where individuals at higher elevation do not reach larger body size; (ii) phenotypic plasticity; or (iii) even a seasonal life history effect. Seasonality could impact body size if individuals at higher elevations hatch or metamorphose later, so that at any given point in time, a random sampling of individuals would on average be younger and thus smaller than their conspecifics at lower elevations. Yet, such seasonal effects could also be adaptive. Independent from these open questions, our morphological data support that the biology of these animals is influenced by elevation‐associated environmental factors on Montagne d'Ambre, and adaptational responses to these effects are therefore plausible.

### 4.4 A common ecotone, a common substrate for speciation?

Perhaps the most important question raised by our results in the context of diversification mechanisms in Madagascar's biota is whether the weak genetic structure in our target species can be considered as representing an early stage of speciation. That is, will the observed divergences, whether initiated by microallopatry or by adaptive processes, ultimately lead to completed speciation, despite gene flow due to the close proximity or direct spatial contact of the genetic clusters? The significantly different ecological niches detected between genetic clusters of these four species satisfy the first prediction for ecological speciation events (Nosil, 2012), according to which events coinciding with shifts in ecological niche should be detectable at the phylogenetic level. However, it is clear that speciation proceeds along a continuum (De Queiroz, 2007; Nosil, 2012), from complete admixture (one species) to complete isolation (two or more species). In some cases, total reproductive isolation is never achieved, but the lineages formed by the divergence process are nevertheless best addressed as species. For example, Pujolar et al. (2022) found in tropical birds that even divergent taxon pairs at high elevations on different massifs experience gene flow across these apparently substantial barriers. It also is important to acknowledge that in‐depth population genomic approaches will allow novel insights into supposedly well‐understood systems; examples in Madagascar are the studies of Poelstra et al. (2021, 2022), who challenged current classification of mouse lemurs by revealing hybridization with mitochondrial introgression in one case, and lack of admixture in a previously postulated hybrid zone in another.

While it is tempting to invoke ecological speciation along elevational gradients as proximate cause for the formation of such related, parapatric species specialized to different elevations, clear evidence for completion of speciation under such conditions is rare, if not absent. In several case studies, close examination of related species distributed at different elevations has revealed that in fact they do not represent direct sister species (Caro et al., 2013), or reconstructed complex scenarios of vicariant speciation and secondary contact upon range extension (e.g. Rasolonjatovo et al., 2020). Current evidence supports a strong role for topographic heterogeneity in diversification and species formation (Badgley et al., 2017), but it is likely that the high biodiversity of many montane regions originated by the interplay of multiple evolutionary mechanisms (Brown et al., 2014; Rahbek et al., 2019). The “mountain‐geobiodiversity hypothesis” of Mosbrugger et al. (2018) proposes that montane biodiversity originates by the combined influences of (i) steep ecological gradients allowing adaptation to distinct niches and immigration of pre‐adapted lineages, (ii) past climate fluctuations causing repeated cycles of connectivity and isolation as drivers of divergence, and (iii) highly rugged terrain providing refugia and short migration distances into favorable habitats during climatic change (Muellner‐Riehl, 2019). From a mechanistic perspective, He et al. (2019) have recently proposed the mixing‐isolation‐mixing (MIM) model to explain how complete speciation can be achieved in geographical settings that allow for intermittent cycles of isolation and gene flow, when the periods of isolation are insufficient to complete speciation under conditions of no gene flow. Damasceno et al. (2014) elaborated the Vanishing Refuge Model that combines climate‐driven habitat fragmentation and exposure to new environments, and thus integrates both vicariance and divergent selection.

It is likely that in Madagascar, and specifically in the Montagne d'Ambre system, a similarly complex mechanism is at play. Habitat fragmentation or naturally occurring habitat discontinuities such as in *M. bellyi* may trigger the origin of genetic structure, and the genetic clusters over time may diverge further through adaptive processes. However, current evidence from related, sympatric or parapatric species pairs or phylogroups of amphibians and reptiles on Montagne d'Ambre provides little evidence for completion of speciation through this mechanism alone. *Mantidactylus ambreensis* and *M. ambony* likely evolved in allopatry, with *M. ambreensis* colonizing low elevations of the massif only later (Rasolonjatovo et al., 2020). In frogs of the *Stumpffia hara* group, the high‐elevation Montagne d'Ambre endemic *S. bishopi* is sister to all other species of the group, while the more widespread *S. megsoni* colonized the western slope of the massif secondarily (Rakotoarison et al., 2022). In arboreal *Cophyla* frogs, the three Montagne d'Ambre endemic species are not each other's closest relatives, which may reflect a convoluted history of independent colonizations as well (Rakotoarison et al., 2015). Dwarf geckos (*Lygodactylus*) are represented by three microendemic and one more widespread species in Montagne d'Ambre, but the microendemics do not appear to be each other's closest relatives (Vences et al., 2022). Other cases of sympatric Montagne d'Ambre endemics either turned out to be synonyms upon closer examination (*Brookesia antakarana* and *B. ambreensis*; Scherz et al., 2018) or have more widespread distributed haplotypes probably due to a complex history of hybridization and introgression as in *Phelsuma* day geckos (Gehring et al., 2022). Only two other species of *Stumpffia* (*S. angeluci* and *S. maledicta*) so far qualify as a genuinely microendemic pair of sister species that may have formed in this small‐scale geographical setting (Rakotoarison et al., 2017), and we are currently investigating this clade further.

In conclusion, the close study of the herpetofauna in the Montagne d'Ambre system herein and in our previous work (Gehring et al., 2022; Rakotoarison et al., 2015, 2022; Rasolonjatovo et al., 2020, 2022; Scherz et al., 2018, 2020) has revealed parallelisms between community composition and genetic differentiation across the mountain's elevational gradient. The existence of a main turnover separating low‐elevation from higher elevation species assemblages supports the importance of elevational adaptation in generating the astonishing species diversity in Madagascar's rainforests. The origin or maintenance of the observed population genetic divergences may also be substantially influenced by elevation, and appears to coincide well with the community turnover, suggesting a common substrate for these differences. However, these divergences are not sufficiently stark or complete to invoke ecological adaptation in sympatry or parapatry as the main driver for the completion of speciation in the amphibians and reptiles of Montagne d'Ambre; additional mechanisms are likely key to generating and maintaining this diversity.

## AUTHOR CONTRIBUTIONS

**Mark D. Scherz:** Conceptualization (equal); formal analysis (equal); resources (equal); writing – original draft (equal). **Robin Schmidt:** Formal analysis (equal); investigation (equal); writing – review and editing (equal). **Jason L. Brown:** Formal analysis (equal); writing – review and editing (equal). **Julian Glos:** Formal analysis (equal); writing – review and editing (equal). **Ella Z. Lattenkamp:** Resources (equal); writing – review and editing (equal). **Zafimahery Rakotomalala:** Resources (equal). **Andolalao Rakotoarison:** Resources (equal). **Ricky T. Rakotonindrina:** Resources (equal). **Onja Randriamalala:** Resources (equal). **Achille P. Raselimanana:** Resources (equal). **Safidy M. Rasolonjatovo:** Resources (equal). **Fanomezana M. Ratsoavina:** Resources (equal). **Jary H. Razafindraibe:** Resources (equal). **Frank Glaw:** Writing – review and editing (equal). **Miguel Vences:** Conceptualization (lead); formal analysis (equal); funding acquisition (equal); writing – original draft (equal).

## CONFLICT OF INTEREST STATEMENT

The authors declare no conflicts of interest.

## Supporting information


Appendix S1.

## Data Availability

All newly determined Sanger sequences were submitted to GenBank (accession numbers MN628333–MN628413, OM818671–OM818815). The full alignment of all sequences (Sanger and Illumina) is available in fasta and tsv formats from Figshare under DOI 10.6084/m9.figshare.14618175. Under the same Figshare DOI, also the full microsatellite libraries in table format are available.
