# Supplementary material for: Repeated divergence of amphibians and reptiles across an elevational gradient in northern Madagascar
Source: Ecol Evol. 2023 Mar 16;13(3):e9914. doi: 10.1002/ece3.9914 (PMC10019947; doi:10.1002/ece3.9914)
Supplement: Supplementary file 1 — Appendix S1. [file ECE3-13-e9914-s001.docx]

**Scherz et al.: Repeated divergence of amphibians and reptiles across an elevational gradient in northern Madagascar**

**Supplementary Materials**

Supplementary methods

*Microsatellite library development – wet lab*: For each species, digestion of DNA took place in three separate reactions with the restriction enzymes AluI, RsaI, and Hpy166II, and products were combined in equal amounts after heat inactivation of the restriction enzymes. The blunt ends were adenylated (+A) with Klenow (exo-) and dATP, and after heat inactivation of the Klenow (exo-), the reactions were supplemented with ATP to 1 mM and an Illumina Y-adaptor was ligated with T4 DNA ligase. Enrichment of the fragments for microsatellites took place by hybridization to, and magnetic capture of, biotinylated repeat probes (representing two unique dimers, five unique trimers, seven unique tetramers and two unique pentamers). This was followed by amplification and barcoding by PCR, and sequencing on an Illumina MiSeq instrument (2 × 250 bp paired reads)

*Microsatellite genotyping protocol*: Microsatellites were amplified following the nested protocol of Schuelke (2000) modified to use, rather than an M13 sequence, the Illumina sequencing primer sequence (ACACTCTTTCCCTACACGACGCTCTTCCGATCT) as linker, i.e., this sequence preceded all forward primers and was included as a FAM-, NED- or HEX-labelled linker in the PCR. The amplification protocol consisted of 15 min of initial denaturation at 94°C, 30 cycles of 94°C (30 s), 60°C (45 s), 72°C (45 s), followed by 8 cycles of 94°C (30 s), 53°C (45 s), 72°C (45 s), and a final elongation step of 10 min at 72°C. PCR products were diluted once with 15 μl of RNase-free water, 15 μl of Genescan 500–ROX size standard (Applied Biosystems) added to 1 μl of each diluted product, and fragment analysis was performed on an ABI 3130xl Genetic Analyzer. Three markers of different product sizes and labelled with FAM, NED, and HEX were combined in each run.


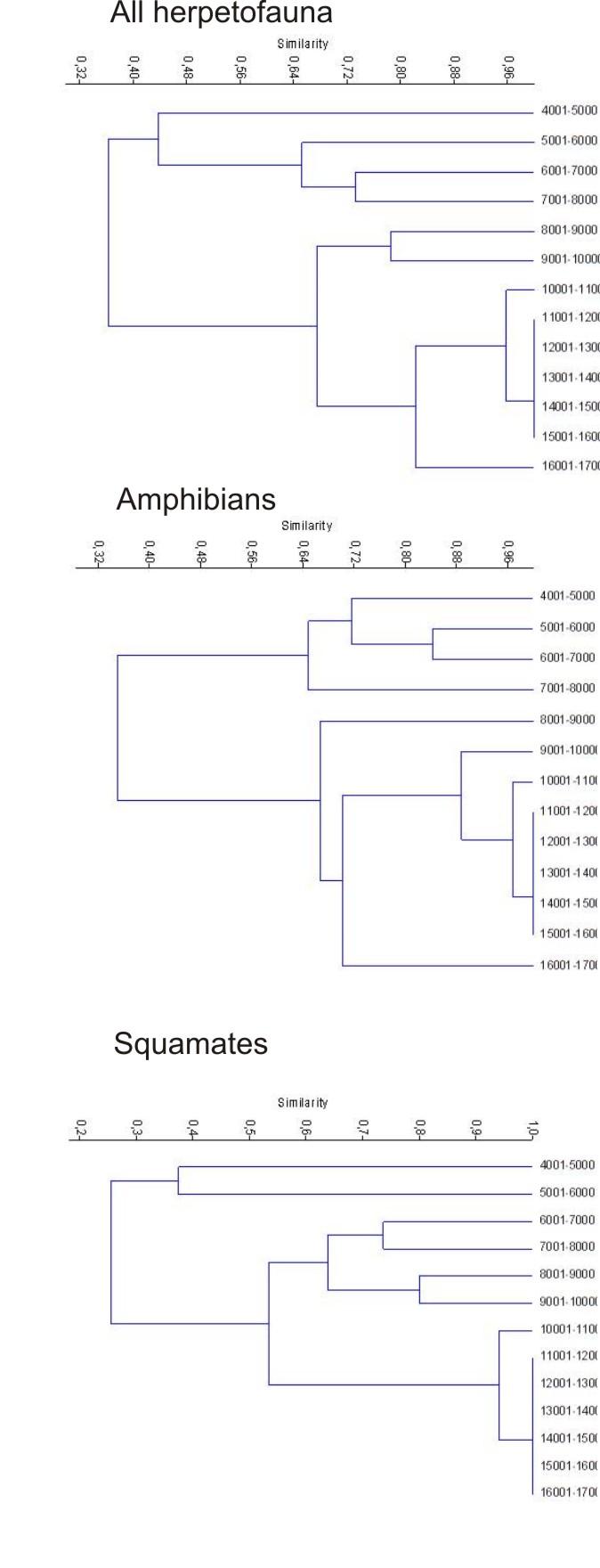


**Supplementary Figure S1**. Community similarity between different 1 km parts of the transect.


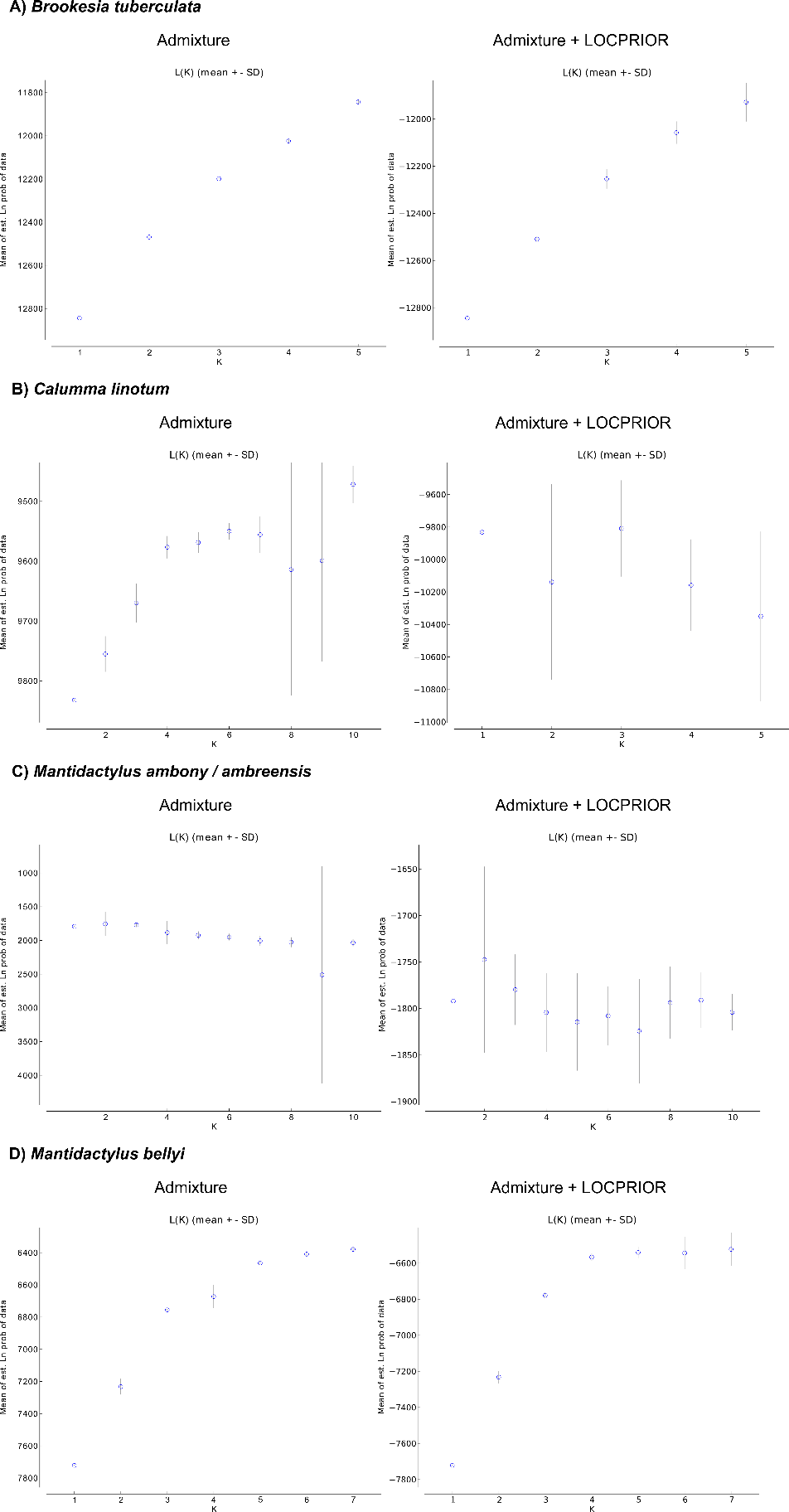


**Supplementary Figure S2.** Likelihood of K and scattering of STRUCTURE replicate runs (admixture model with and without LOCPRIOR) for A) *Brookesia tuberculata* B) *Calumma linotum*, C) *Mantidactylus ambony / M. ambreensis*, and D) *Mantidactylus bellyi.*


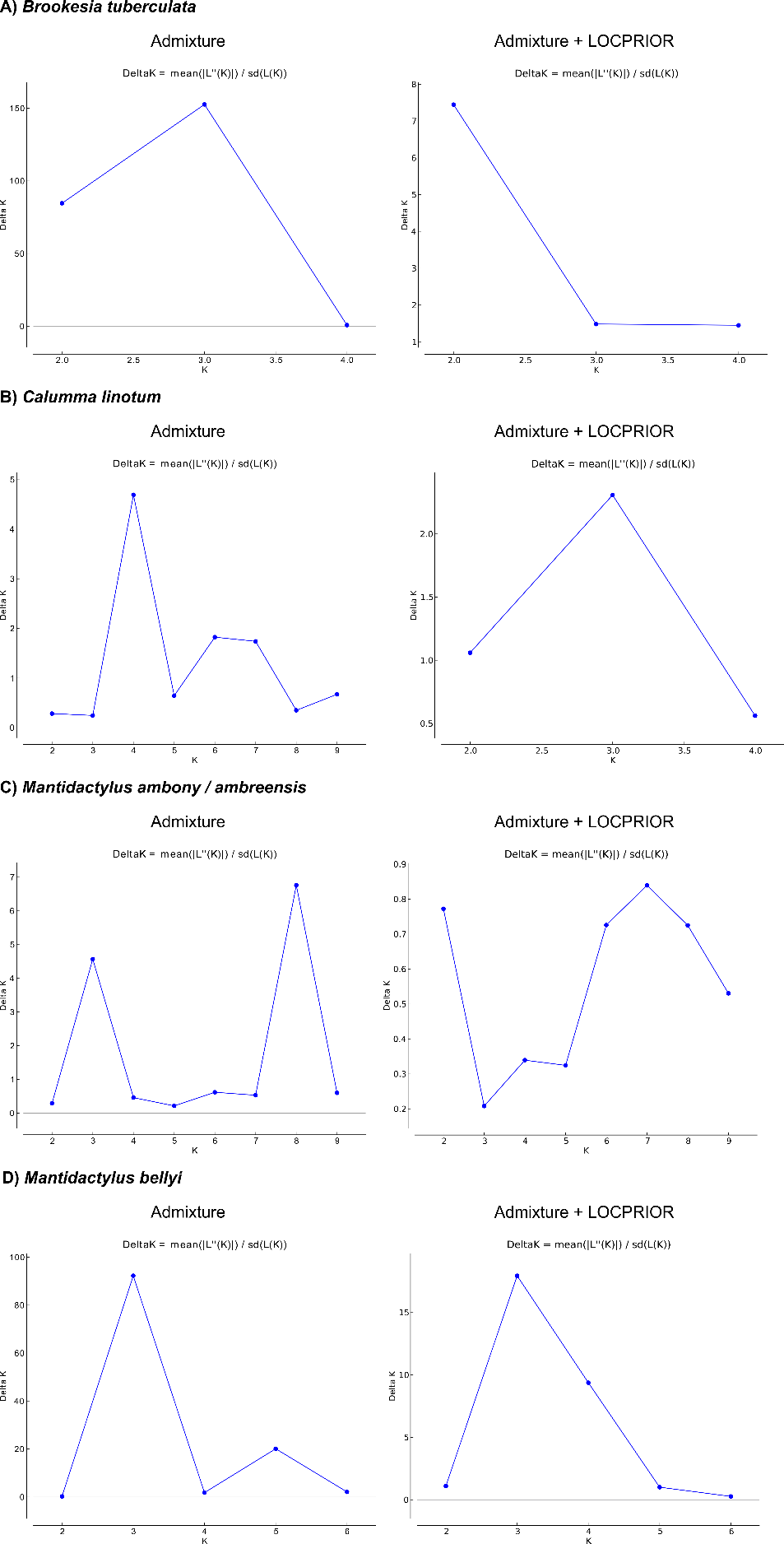


**Supplementary Figure S3**. ∆K graph for determination of the most probable K in STRUCTURE runs (admixture model with and without LOCPRIOR) for A) *Brookesia tuberculata*, B) *Calumma linotum*, C) *Mantidactylus ambony / M. ambreensis*, and D) *Mantidactylus bellyi*.


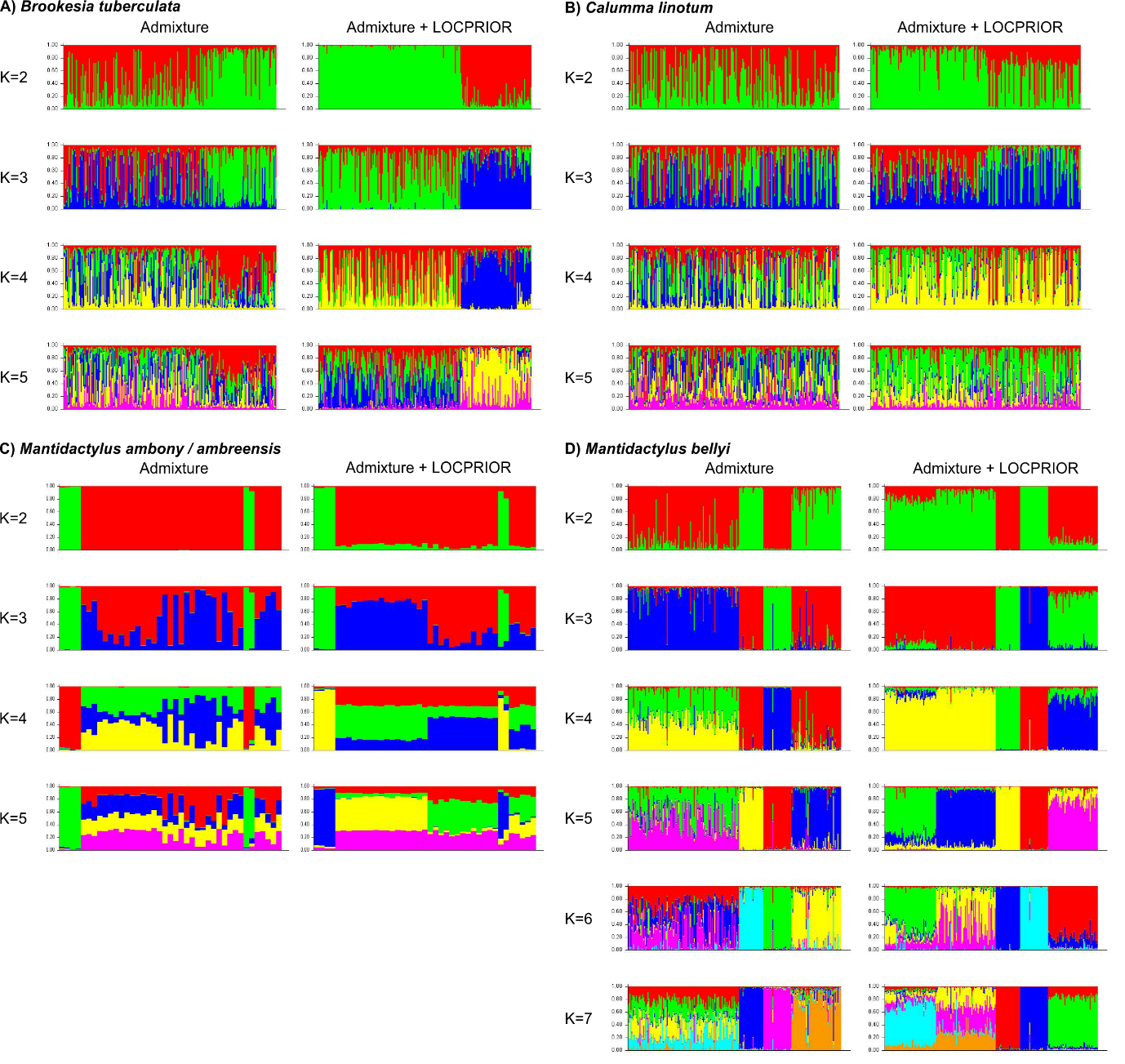


**Supplementary Figure S4**. Genetic cluster assignment of the sampled populations of A) *Brookesia tuberculata*, B) *Calumma linotum*, C) *Mantidactylus ambony / M. ambreensis*, and D) *Mantidactylus bellyi*. Note that the colors are assigned to clusters randomly (“label switching”) in each STRUCTURE run (admixture model with and without LOCPRIOR) and are not comparable among graphs.


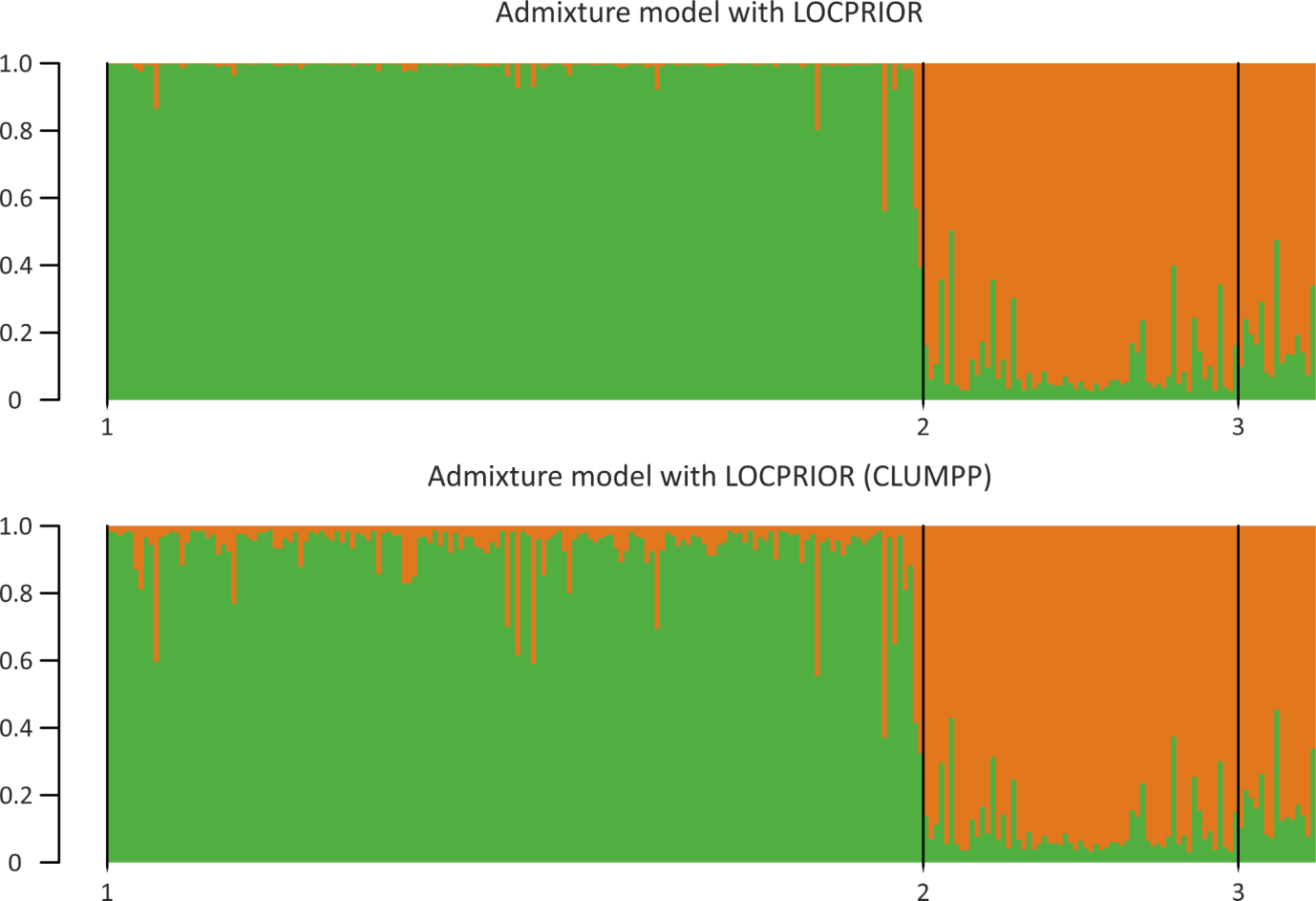


**Supplementary Figure S5**. Plots of genetic assignment of samples of *Brookesia tuberculata* based on analysis of microsatellite data (detailed view of plot shown in Fig. 2) with an admixture model with LOCPRIOR, under regular analysis with STRUCTURE and following the CLUMPP procedure. The LOCPRIOR was set based on ad-hoc geographical groups defined by a sampling gap between groups 1 and 2, and separating group 3 that corresponds to the western slope of Montagne d'Ambre.


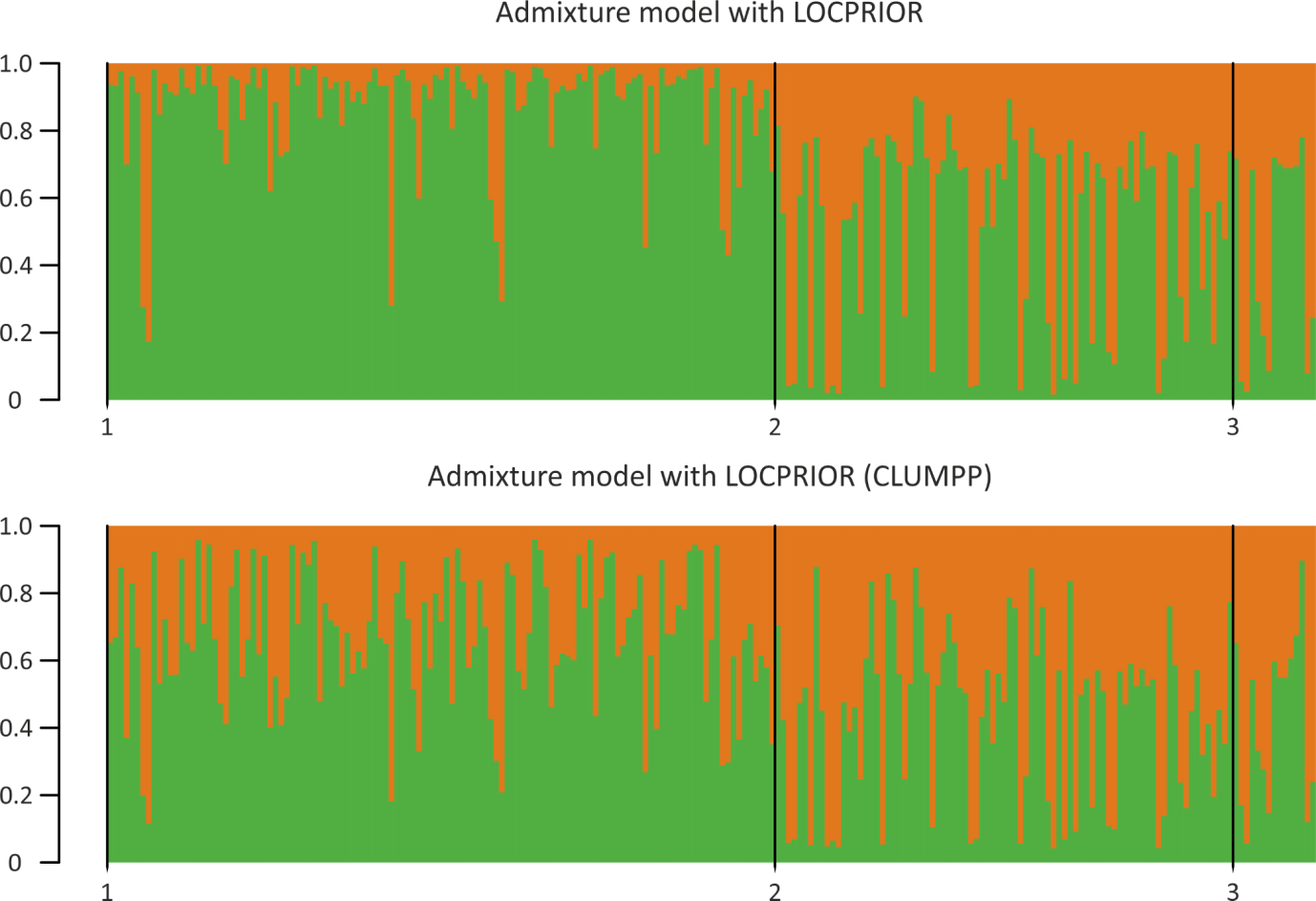


**Supplementary Figure S6**. Plots of genetic assignment of samples of *Calumma linotum* based on analysis of microsatellite data with an admixture model with LOCPRIOR, under regular analysis with STRUCTURE (detailed view of plot shown in Fig. 2) and following the CLUMPP procedure. The LOCPRIOR was set based on ad-hoc geographical groups defined by a difference in mitochondrial haplotype distribution between groups 1 and 2, and separating group 3 that corresponds to the western slope of Montagne d'Ambre.


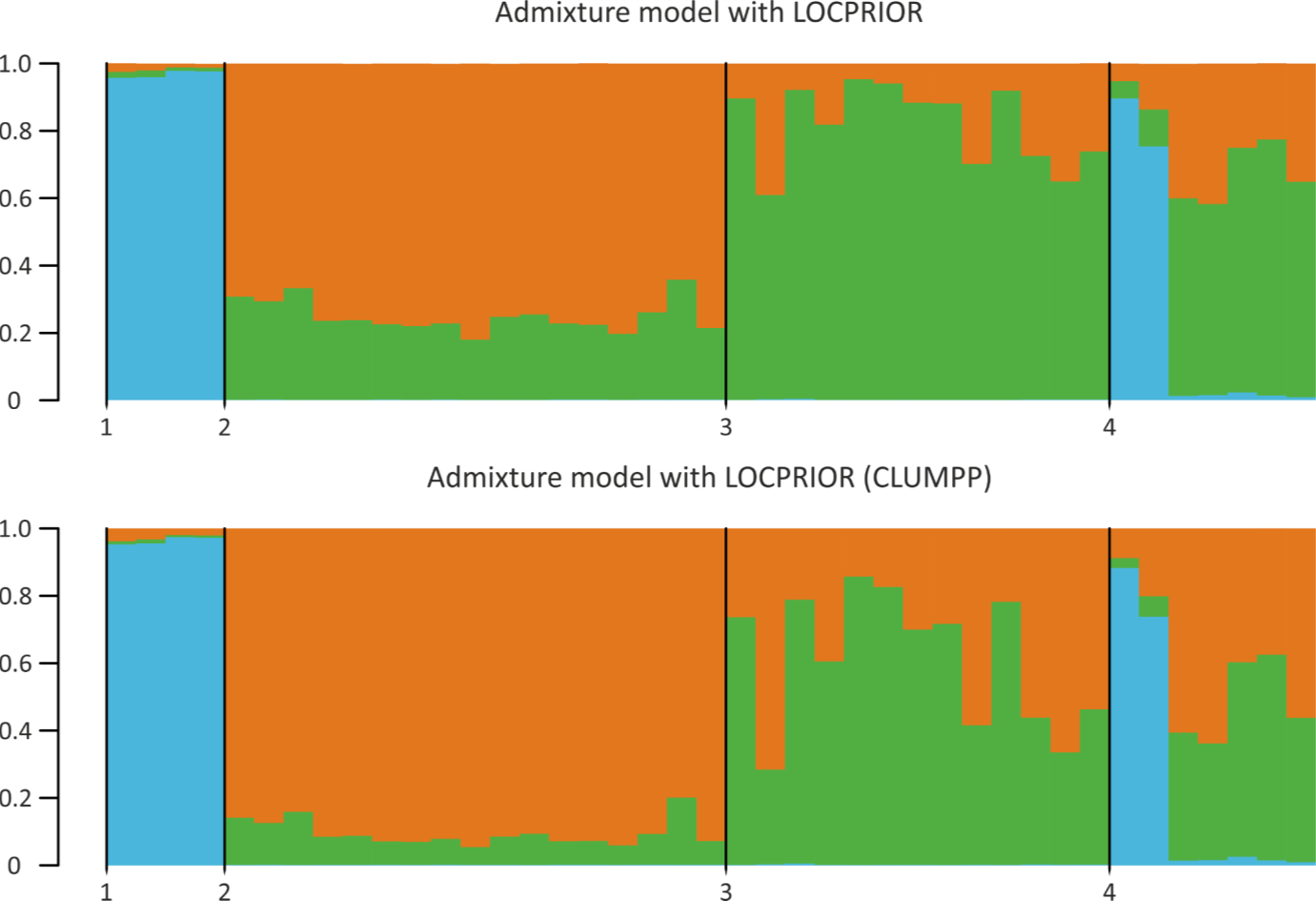


**Supplementary Figure S7.** Plots of genetic assignment of samples of the *Mantidactylus ambony / M. ambreensis* microsatellite dataset with an admixture model with LOCPRIOR, under regular analysis with STRUCTURE (detailed view of plot shown in Fig. 2) and following the CLUMPP procedure. The LOCPRIOR was set based on ad-hoc geographical groups defined by geographical proximity of sampling localities. The blue cluster corresponds to samples of *M. ambreensis* while green and orange clusters correspond to weakly defined clusters of *M. ambony*.

*
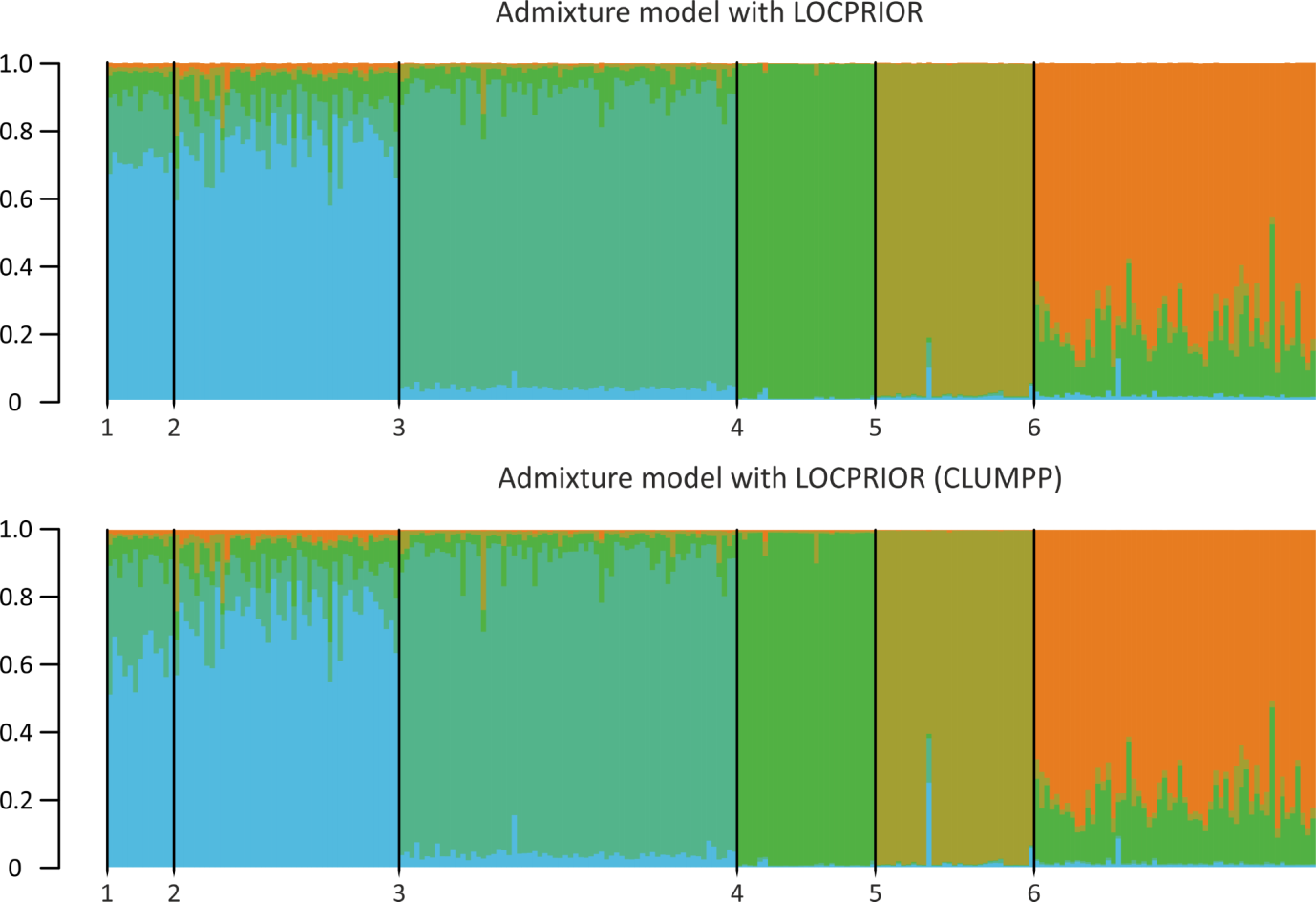
*

**Supplementary Figure S8**. Plots of genetic assignment of samples of the *Mantidactylus bellyi* microsatellite dataset with an admixture model with LOCPRIOR, under regular analysis with STRUCTURE (detailed view of plot shown in Fig. 2) and following the CLUMPP procedure. The LOCPRIOR was set based on ad-hoc geographical groups defined by geographical proximity of sampling localities.


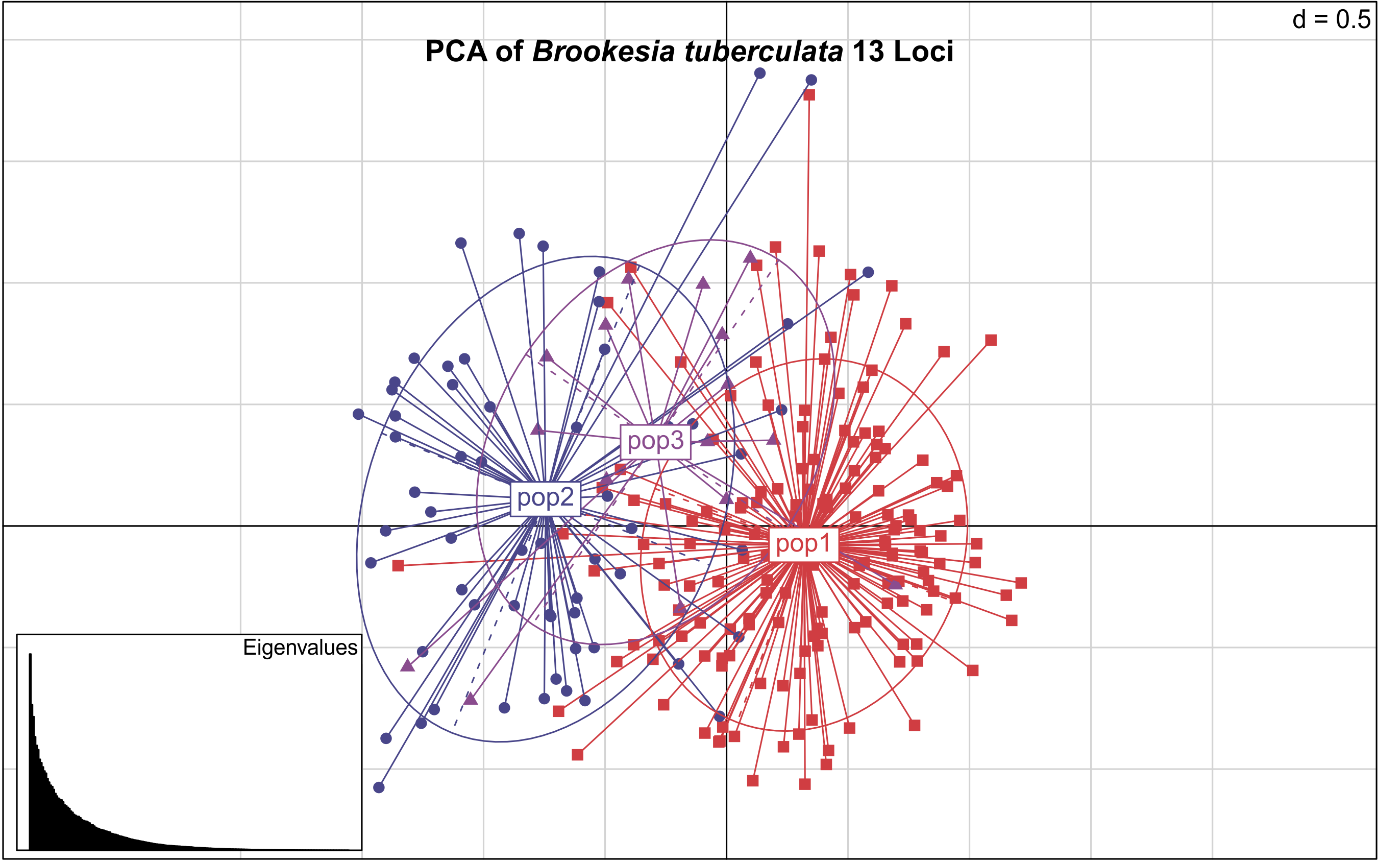


**Supplementary Figure S9**. Principal component analysis (PCA) of genotype data from 13 microsatellite loci for 3 locality groups of *B. tuberculata*. The scatterplot shows samples (each dot corresponding to one sample) according to their scores for PC1 (X-axis) and PC2 (Y-axis). Samples are colored according to a-priori locality groups (pop1 = locality group 1; pop2 = locality group 2; pop3 = locality group 3). Circles represent inertia ellipses.


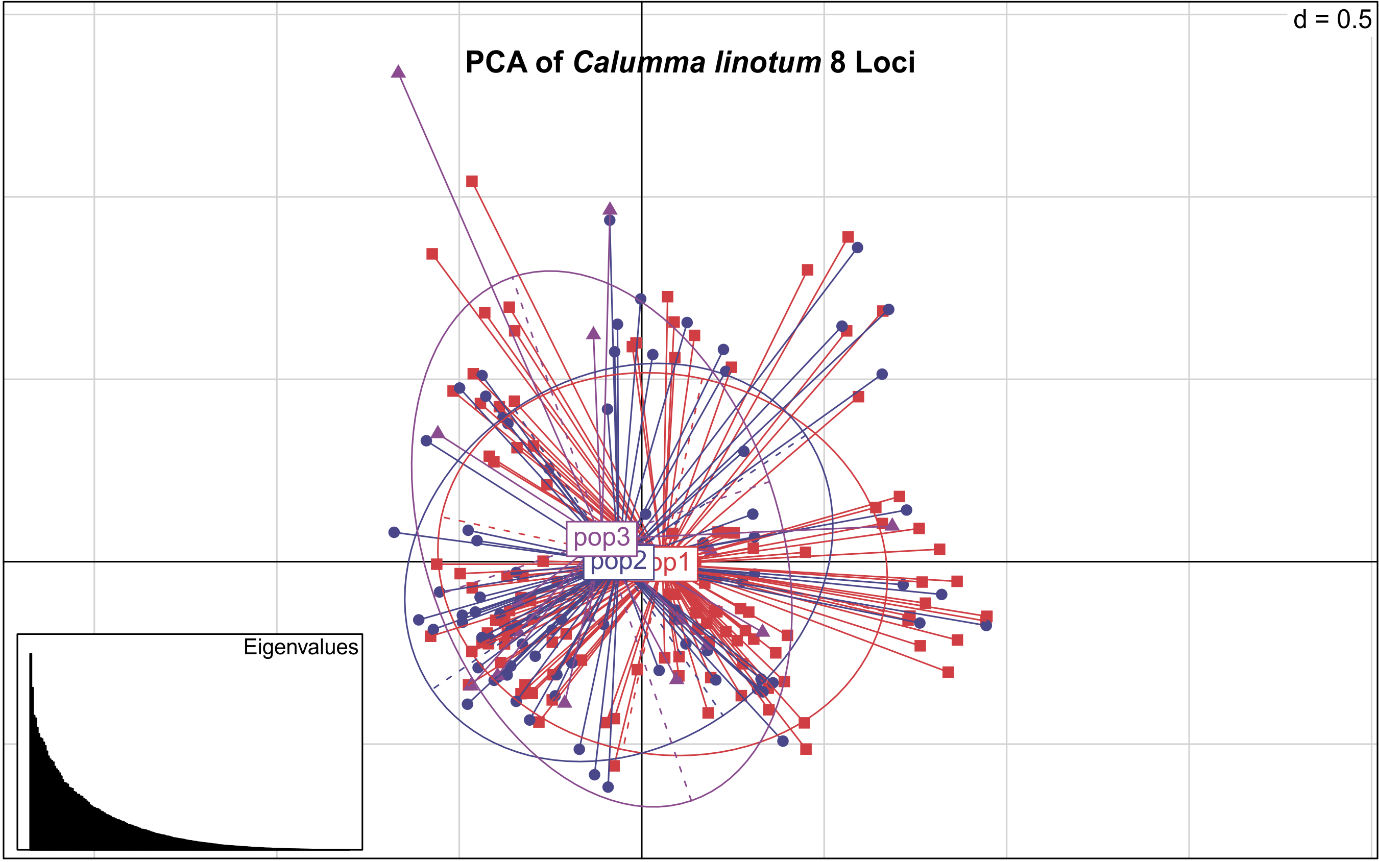


**Supplementary Figure S10**. Principal component analysis (PCA) of genotype data from 8 microsatellite loci for 3 locality groups of *C. linotum*. The scatterplot shows samples (each dot corresponding to one sample) according to their scores for PC1 (X-axis) and PC2 (Y-axis). Samples are colored according to a-priori locality groups (pop1 = locality group 1; pop2 = locality group 2; pop3 = locality group 3). Circles represent inertia ellipses.


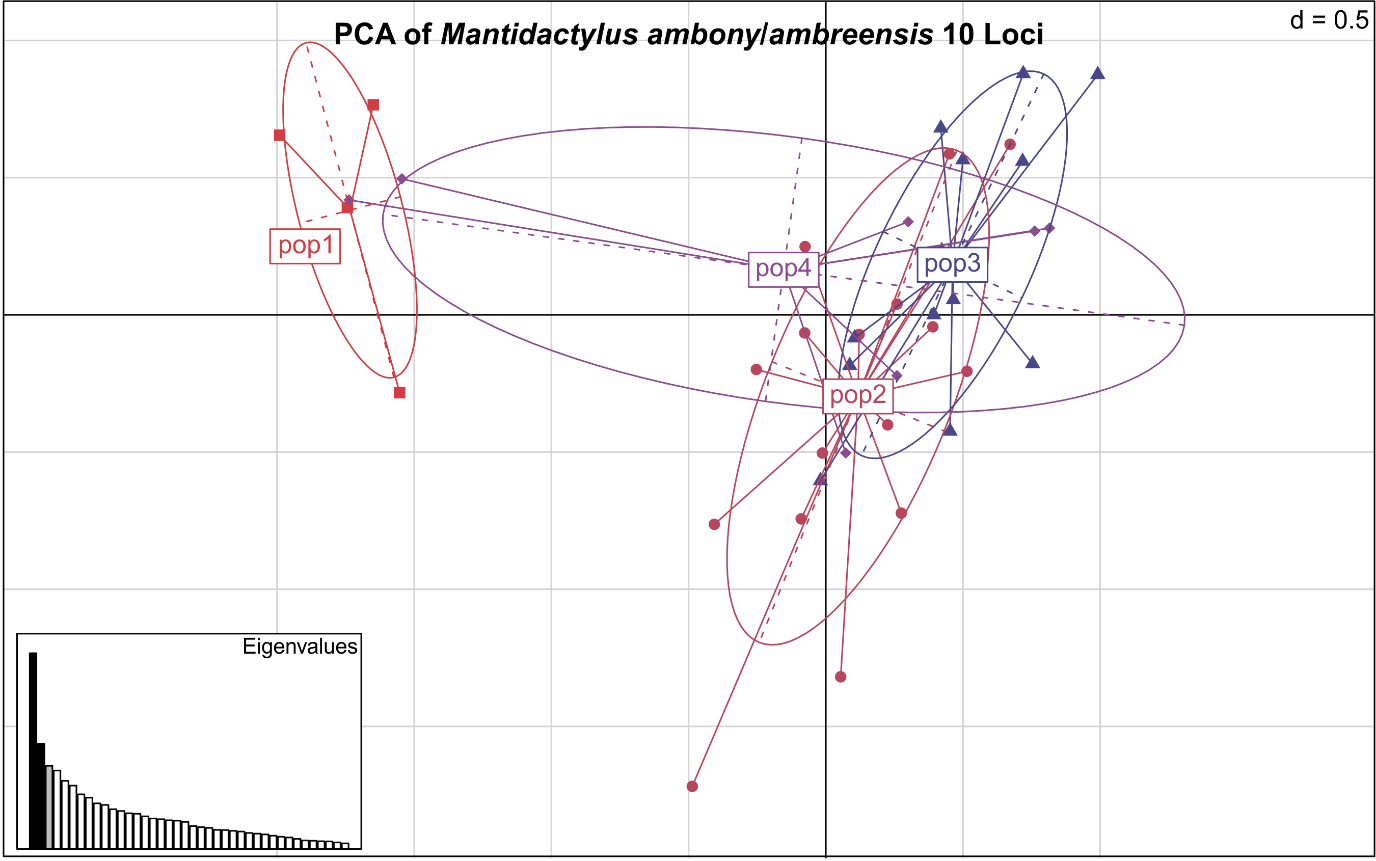


**Supplementary Figure S11**. Principal component analysis (PCA) of genotype data from 10 microsatellite loci for 4 locality groups of *M. ambony / M. ambreensis*. The scatterplot shows samples (each dot corresponding to one sample) according to their scores PC1 (X-axis) and PC2 (Y-axis). Samples are colored according to a-priori locality groups (pop1 = locality group 1; pop2 = locality group 2; pop3 = locality group 3; pop4 = locality group 4). Circles represent inertia ellipses. The cluster on the left, containing four individuals of locality group 1 (pop1) and two individuals of locality group 4 (pop4) represents *M. ambreensis,* the other correspond to *M. ambony.* See Supplementary Figure S12 for a PCA plot containing only *M. ambony.*


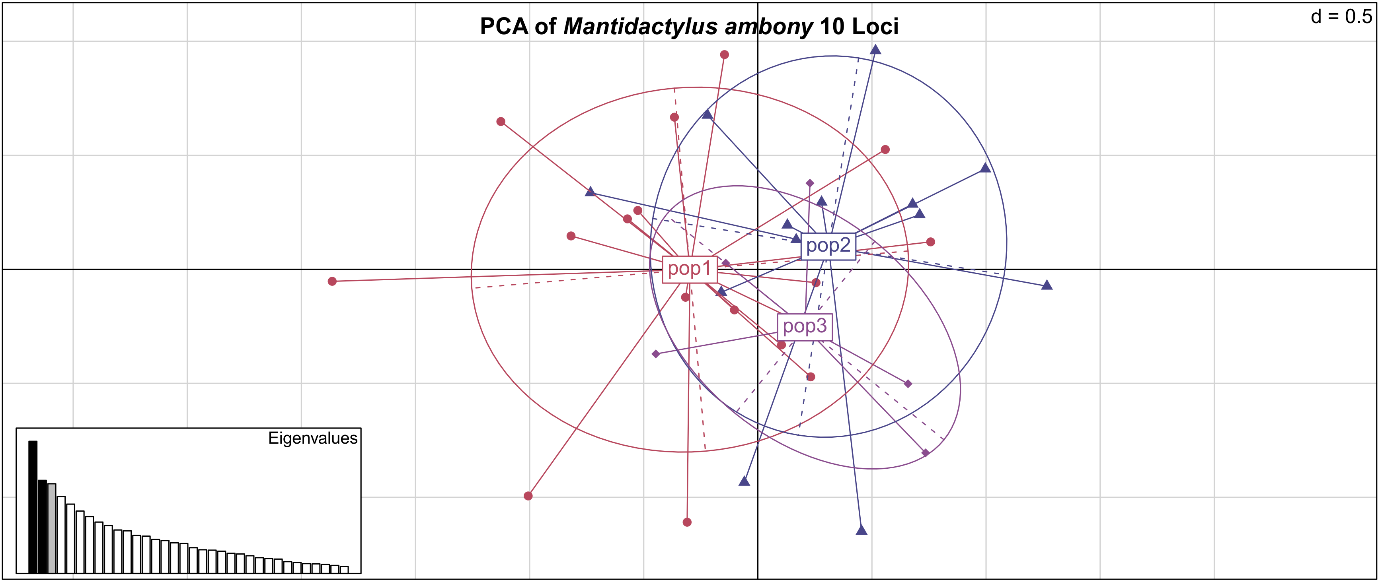


**Supplementary Figure S12**. Principal component analysis (PCA) of genotype data from 10 microsatellite loci for 3 locality groups of *M. ambony*. The scatterplot shows samples (each dot corresponding to one sample) according to their scores for PC1 (X-axis) and PC2 (Y-axis). Samples are colored according to a-priori locality groups (pop1 = locality group 2; pop2 = locality group 3; pop3 = locality group 4). Circles represent inertia ellipses.


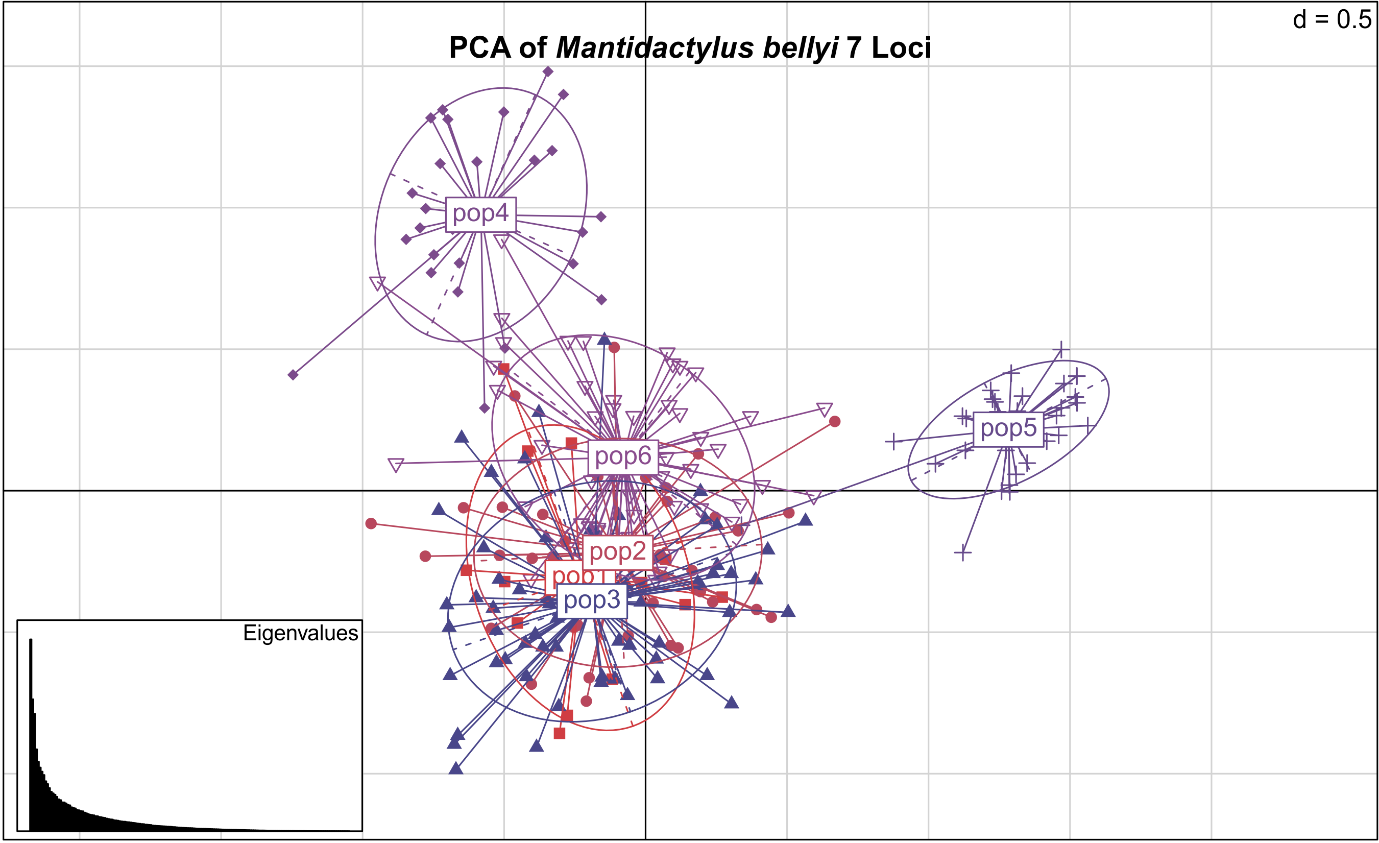


**Supplementary Figure S13**. Principal component analysis (PCA) of genotype data from 7 microsatellite loci for 6 locality groups of *M. bellyi*. The scatterplot shows samples (each dot corresponding to one sample) according to their scores for PC1 (X-axis) and PC2 (Y-axis). Samples are colored according to a-priori locality groups (pop1 = locality group 1; pop2 = locality group 2; pop3 = locality group 3; pop4 = locality group 4; pop5 = locality group 5; pop6 = locality group 6). Circles represent inertia ellipses.


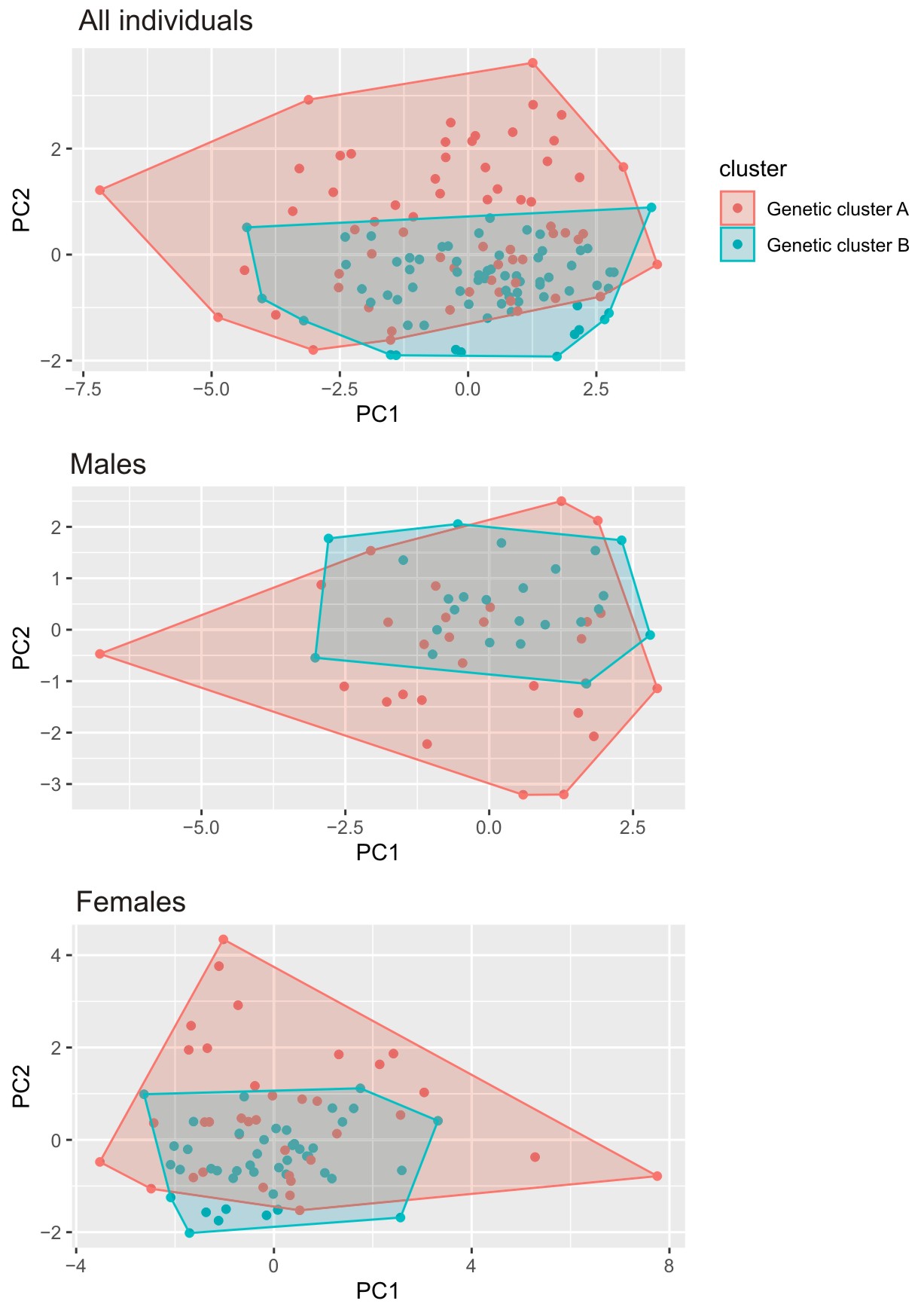


**Supplementary Figure S14**. Scatterplot of factor loadings from PC1 and PC2 of a PCA of size-corrected morphometric data of *C. linotum,* separated for genetic clusters A and B, for all individuals and separately for males and females.


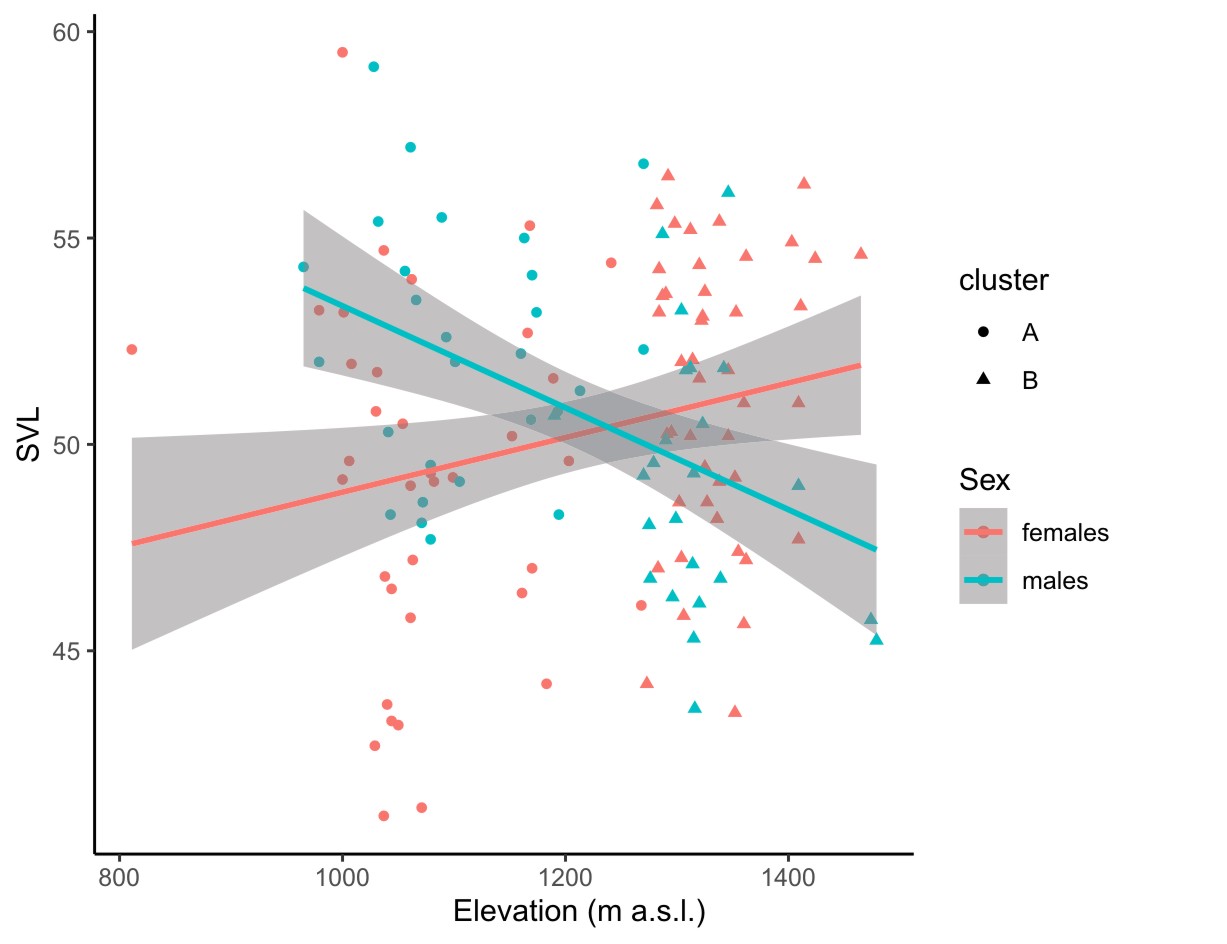


**Supplementary Figure S15**. Body size of *Calumma linotum* relative to elevation, including data from males (also included in Fig. 3) and females, suggesting a possible inverse trend in both sexes. Note however that it cannot be excluded that the small-sized females from lower elevations are subadults, and the female trend is therefore not further discussed herein.

**Supplementary Table S1**. List of forward (Fwd) and reverse (Rev) primers for newly established microsatellite markers for *Brookesia tuberculata, Calumma linotum, Mantidactylus ambreensis* and *M. bellyi*. Repeat counts are from the initial library; numbers and length ranges of alleles, as well as % missing data, refer to the entire set of 234, 219, 41, and 236 samples genotyped, respectively. The adapters are shown in square brackets (note that the adapter used for *M. bellyi* differs by lacking the terminal nucleotide).

| Marker | 5'-3' primer sequence | Repeat | N alleles | Length  range | % Missing  data |
| --- | --- | --- | --- | --- | --- |
| *B. tuberculata* |  |  |  |  |  |
| Btub_10333 | Fwd: [ACACTCTTTCCCTACACGACGCTCTTCCGATCT]GCCTCCAAGTCAGCATCTTAAC  Rev: AGGTTCACAGCCACTAACCTAG | AGAT_8_ | 13 | 284-336 | 0 |
| Btub_11367 | Fwd: [ACACTCTTTCCCTACACGACGCTCTTCCGATCT]AAAGCAGAACTGAAGAGCCC  Rev: CTCAGACTGAATTTCCACTCGG | AAAG_7_ | 9 | 230-262 | 3.8 |
| Btub_13074 | Fwd: [ACACTCTTTCCCTACACGACGCTCTTCCGATCT]TGGCGACCACTGCATTAGATAG  Rev: CTCGAACTGCTGACCTATTGAC | ATCC_8_ | 17 | 154-226 | 2.6 |
| Btub_19069 | Fwd: [ACACTCTTTCCCTACACGACGCTCTTCCGATCT]GTACCTAAACACAGCTGCTTGG  Rev: GGACAACCTGCTGAATGGTATC | AGAT_7_ | 24 | 200-308 | 0 |
| Btub_24194 | Fwd: [ACACTCTTTCCCTACACGACGCTCTTCCGATCT]ACGAGGGATTACTGTAGCTGAG  Rev: GGCCTTCCATTCAGTGAGTTC | AAAG_10_ | 18 | 192-252 | 2.1 |
| Btub_27139 | Fwd: [ACACTCTTTCCCTACACGACGCTCTTCCGATCT]GCAAGGGTGACTCTTATCCATTC  Rev: GATAGCCCTCAAACTGTCATACC | AGAT_14_ | 20 | 171-255 | 0.9 |
| Btub_33126 | Fwd: [ACACTCTTTCCCTACACGACGCTCTTCCGATCT]CAGAGTATTGGGAATTGGGCG  Rev: GAAGCTTAGCCTGGAGAATAGC | ACAT_11_ | 26 | 180-282 | 1.7 |
| Btub_37722 | Fwd: [ACACTCTTTCCCTACACGACGCTCTTCCGATCT]CTCGAACTGCTGACCTATTGAC  Rev: AAGCGTCCTAAGTTTGGCAAC | AGAT_9_ | 11 | 184-228 | 6.8 |
| Btub_39046 | Fwd: [ACACTCTTTCCCTACACGACGCTCTTCCGATCT]TACACAGAGGCTACACAGGATG  Rev: GGGTTACATCTCCATTCCTGTC | ACAT_7_ | 17 | 349-409 | 7.3 |
| Btub_40780 | Fwd: [ACACTCTTTCCCTACACGACGCTCTTCCGATCT]CTCCTGTTCATGTGGTAGAAGC  Rev: GCTGGTACTTGATGACCGATTG | AGAT_12_ | 22 | 262-346 | 0.9 |
| Btub_41087 | Fwd: [ACACTCTTTCCCTACACGACGCTCTTCCGATCT]CTTTCAGCCAGTGTAATCAGGG  Rev: CCATTGATGAAACGAGAGGGTG | AGAT_10_ | 16 | 164-220 | 0 |
| Btub_59292 | Fwd: [ACACTCTTTCCCTACACGACGCTCTTCCGATCT]GAAGCTTAGCCTGGAGAATAGC  Rev: CAGAGTATTGGGAATTGGGCG | AGAT_11_ | 24 | 180-260 | 0.4 |
| Btub_67320 | Fwd: [ACACTCTTTCCCTACACGACGCTCTTCCGATCT]TCCCACTTGAATAGGTACCAGC  Rev: ATAGGTGGGCAGATAGACAGAC | AGAT_7_ | 11 | 212-252 | 7.3 |
| *C. linotum* |  |  |  |  |  |
| Clin_23074 | Fwd: [ACACTCTTTCCCTACACGACGCTCTTCCGATCT]GCACTCATGTCTCGAACTGATG  Rev: TGAATTCCTCCTCTTCCTTGGG | AAAC10 | 11 | 344-380 | 4.1 |
| Clin_33986 | Fwd: [ACACTCTTTCCCTACACGACGCTCTTCCGATCT]ACCCTAGATTTGGACAGGACTG  Rev: CTAGTGGGCAAAGAATGGATGG | AGAT12 | 37 | 143-295 | 5.9 |
| Clin_4572 | Fwd: [ACACTCTTTCCCTACACGACGCTCTTCCGATCT]GGCACATGACCATTAACTTCCC  Rev: CTAACTTGGTCTTGGCATTCCC | AGAT10 | 21 | 186-262 | 8.2 |
| Clin_488 | Fwd: [ACACTCTTTCCCTACACGACGCTCTTCCGATCT]ACCCTGCTCAGTACTTATTCCC  Rev: CTTCATGGCTGGTTGACTTCTC | AAAG12 | 30 | 382-492 | 2.3 |
| Clin_50973 | Fwd: [ACACTCTTTCCCTACACGACGCTCTTCCGATCT]CCTTATGTTCTTGGAGTTGGGC  Rev: TGATGTTAGCCACCCTGAGTAG | AGAT10 | 25 | 172-280 | 1.4 |
| Clin_615 | Fwd: [ACACTCTTTCCCTACACGACGCTCTTCCGATCT]CTTCATGGCTGGTTGACTTCTC  Rev: ACCCTGCTCAGTACTTATTCCC | AAAG10 | 30 | 385-505 | 3.2 |
| Clin_733 | Fwd: [ACACTCTTTCCCTACACGACGCTCTTCCGATCT]GACCTGAAATGCATAGTGGGTG  Rev: CATAACTCTGACCCACTCGTTG | AGAT14 | 38 | 145-328 | 3.2 |
| Clin_870 | Fwd: [ACACTCTTTCCCTACACGACGCTCTTCCGATCT]TGAACTTCAACTCCGAGAAGCC  Rev: GGAGCCATGCCAAGTATTATCC | AAAG14 | 27 | 174-272 | 4.6 |
| *M. ambreensis* |  |  |  |  |  |
| Mamb_11469 | Fwd: [ACACTCTTTCCCTACACGACGCTCTTCCGATCT]CAAACACACCTCCAAGATGACC  Rev: CTCCTGCTGTGATCTCATTAGTG | ACAT13 | 15 | 267-271 | 0 |
| Mamb_12420 | Fwd: ACACTCTTTCCCTACACGACGCTCTTCCGATCTCCTGGATTCTCTGTGTACTTGC  Rev: GCACAGTATAGATGTCCAGCAC | AGAT13 | 9 | 225-277 | 0 |
| Mamb_13883 | Fwd: ACACTCTTTCCCTACACGACGCTCTTCCGATCTCGAATACAGCACCACCATCTAG  Rev: GGCTCTCTGTGTAAGTGATTCC | ACAT10 | 10 | 227-347 | 0 |
| Mamb_15504 | Fwd: ACACTCTTTCCCTACACGACGCTCTTCCGATCTCTGAAAGTCTGCAGTTCAACGG  Rev: AACGGTACCTGGATCATGACTG | ACAT11 | 11 | 138-190 | 0 |
| Mamb_2411 | Fwd: [ACACTCTTTCCCTACACGACGCTCTTCCGATCT]CATGTTACAACCATCCTCCCTG  Rev: CCCTAAAGCCGAAGACCAATTG | ACAT13 | 27 | 307-427 | 0 |
| Mamb_2892 | Fwd: [ACACTCTTTCCCTACACGACGCTCTTCCGATCT]GGAAGTGGTATCAGCAGAGAAC  Rev: CTTCCGTCCATTCACCTTAACC | AGAT15 | 29 | 227-355 | 0 |
| Mamb_3079 | Fwd: [ACACTCTTTCCCTACACGACGCTCTTCCGATCT]CCGAACTGTACTGTGTGGAAAC  Rev: CCCTCCTGTCACAGTATGTATG | ACAT10 | 5 | 278-298 | 7.3 |
| Mamb_4540 | Fwd: [ACACTCTTTCCCTACACGACGCTCTTCCGATCT]CATATGCCTGGACTTGTCACAG  Rev: CCACTTCTCACATGTACACCAC | ACAT13 | 19 | 196-336 | 0 |
| Mamb_8314 | Fwd: [ACACTCTTTCCCTACACGACGCTCTTCCGATCT]CTTTGCCTTTCACCTCCATCTC  Rev: GGCAGACACACCTCAAAGTATG | ACAT10 | 9 | 241-273 | 0 |
| Mamb_9283 | Fwd: [ACACTCTTTCCCTACACGACGCTCTTCCGATCT]CCTTGTGTCAGATGCAGAACAC  Rev: GACAGCAGCACATGTATGATCC | ACAT14 | 23 | 212-308 | 0 |
| *M. bellyi* |  |  |  |  |  |
| Mbell_102671 | Fwd: [ACACTCTTTCCCTACACGACGCTCTTCCGATC]GTTGCTACGAGAGGATTGTGTG  Rev: ACCACTATAGCTGTGTCACCTG | AGAT11 | 29 | 199-319 | 0.4 |
| Mbell_158602 | Fwd: [ACACTCTTTCCCTACACGACGCTCTTCCGATC]GCAGAGAACTTGTTGGCCATAC  Rev: GACCAGCTTCATATTTCTGGCC | ATCC12 | 25 | 129-191 | 1.7 |
| Mbell_16089 | Fwd: [ACACTCTTTCCCTACACGACGCTCTTCCGATC]CAACGACTGCCAGATTTCCTAG  Rev: TGCCCTAGTGAGACCTTTGAAC | ACAT15 | 19 | 254-338 | 1.7 |
| Mbell_19325 | Fwd: [ACACTCTTTCCCTACACGACGCTCTTCCGATC]CTTCTTTAGCAAGGCAGTGGTC  Rev: TGATATTAGTCTCTGGCCTGGC | AGAT14 | 21 | 290-382 | 14.4 |
| Mbell_27880 | Fwd: [ACACTCTTTCCCTACACGACGCTCTTCCGATC]CACTATGCATGACTGGCACTAG  Rev: ATCGTCTCCTGTGTCCTATGTG | AGAT12 | 19 | 362-486 | 13.6 |
| Mbell_31964 | Fwd: [ACACTCTTTCCCTACACGACGCTCTTCCGATC]CTTTCTTGACCAACTCCACTCC  Rev: CCCTCAGAAACTGCAGTGAATG | ACAG14 | 31 | 370-498 | 18.2 |
| Mbell_333 | Fwd: [ACACTCTTTCCCTACACGACGCTCTTCCGATC]GTTCCACTTCTCACGTGTACAC  Rev: CGTGTATGAGCGACTATTCACC | ACAT15 | 38 | 208-320 | 0.8 |

**Supplementary Table S2**. Analysis of the Hardy-Weinberg equilibrium for a) 13 microsatellite loci for three locality groups of *B. tuberculata*; b) eight microsatellite loci for three locality groups of *C. linotum*; c) ten microsatellite loci for four locality groups of *M. ambony / M. ambreensis*; d) seven microsatellite loci for six locality groups of *M. bellyi* (*H_O_* = observed heterozygosity, *H_E_*. = expected heterozygosity, *P_HW_* = Hardy-Weinberg probability test, s.d. = standard deviation, *indicates deviation from Hardy-Weinberg equilibrium (P < 0.05) after Bonferroni correction).

a)

|  | **Locality Group 1** | | | | **Locality Group 2** | | | | **Locality Group 3** | | | |
| --- | --- | --- | --- | --- | --- | --- | --- | --- | --- | --- | --- | --- |
| **Locus** | ***H_O_.*** | ***H_E_.*** | ***P_HW_*** | **s.d.** | ***H_O_.*** | ***H_E_.*** | ***P_HW_*** | **s.d.** | ***H_O_.*** | ***H_E_.*** | ***P_HW_*** | **s.d.** |
| Btub_10333 | 0.739 | 0.812 | 0.16409 | 0.00082 | 0.852 | 0.822 | 0.43989 | 0.00075 | 0.688 | 0.847 | 0.17104 | 0.00080 |
| Btub_11367 | 0.213 | 0.730 | 0.00000* | 0.00000 | 0.169 | 0.648 | 0.00000* | 0.00000 | 0.188 | 0.716 | 0.00000* | 0.00000 |
| Btub_13074 | 0.513 | 0.886 | 0.00000* | 0.00000 | 0.377 | 0.834 | 0.00000* | 0.00000 | 0.333 | 0.887 | 0.00000* | 0.00000 |
| Btub_19069 | 0.497 | 0.910 | 0.00000* | 0.00000 | 0.590 | 0.919 | 0.00000* | 0.00000 | 0.750 | 0.871 | 0.29418 | 0.00083 |
| Btub_24149 | 0.706 | 0.823 | 0.00355 | 0.00006 | 0.650 | 0.819 | 0.00197 | 0.00012 | 0.938 | 0.875 | 0.93713 | 0.00049 |
| Btub_27139 | 0.821 | 0.872 | 0.62537 | 0.00092 | 0.867 | 0.876 | 0.05618 | 0.00033 | 0.813 | 0.802 | 0.86569 | 0.00096 |
| Btub_33126 | 0.539 | 0.906 | 0.00000* | 0.00000 | 0.650 | 0.893 | 0.00000* | 0.00000 | 0.625 | 0.847 | 0.00333 | 0.00013 |
| Btub_37722 | 0.255 | 0.862 | 0.00000* | 0.00000 | 0.153 | 0.768 | 0.00000* | 0.00000 | 0.143 | 0.836 | 0.00000* | 0.00000 |
| Btub_39046 | 0.689 | 0.788 | 0.15839 | 0.00063 | 0.364 | 0.556 | 0.00061* | 0.00007 | 0.786 | 0.873 | 0.07143 | 0.00030 |
| Btub_40780 | 0.406 | 0.816 | 0.00000* | 0.00000 | 0.295 | 0.843 | 0.00000* | 0.00000 | 0.500 | 0.893 | 0.00364 | 0.00015 |
| Btub_41087 | 0.274 | 0.387 | 0.00000* | 0.00000 | 0.295 | 0.729 | 0.00000* | 0.00000 | 0.375 | 0.808 | 0.00000* | 0.00000 |
| Btub_59292 | 0.481 | 0.899 | 0.00000* | 0.00000 | 0.574 | 0.893 | 0.00000* | 0.00000 | 0.563 | 0.829 | 0.00067* | 0.00010 |
| Btub_67320 | 0.524 | 0.831 | 0.00000* | 0.00000 | 0.696 | 0.767 | 0.11786 | 0.00058 | 0.500 | 0.823 | 0.00043* | 0.00006 |
| **Mean** | 0.512 | 0.809 | 0.07318 | 0.00019 | 0.502 | 0.798 | 0.04742 | 0.00014 | 0.554 | 0.839 | 0.18058 | 0.00029 |
| **Number of samples** | 157 | | | | 61 | | | | 16 | | | |

b)

|  | **Locality Group 1** | | | | **Locality Group 2** | | | | **Locality Group 3** | | | |
| --- | --- | --- | --- | --- | --- | --- | --- | --- | --- | --- | --- | --- |
| **Locus** | ***H_O_.*** | ***H_E_.*** | ***P_HW_*** | **s.d.** | ***H_O_.*** | ***H_E_.*** | ***P_HW_*** | **s.d.** | ***H_O_.*** | ***H_E_.*** | ***P_HW_*** | **s.d.** |
| Clin_488 | 0.916 | 0.939 | 0.74595 | 0.00034 | 0.900 | 0.945 | 0.21127 | 0.00051 | 0.867 | 0.947 | 0.05407 | 0.00025 |
| Clin_615 | 0.922 | 0.936 | 0.44867 | 0.00087 | 0.938 | 0.948 | 0.70940 | 0.00062 | 0.875 | 0.950 | 0.02414 | 0.00013 |
| Clin_733 | 0.915 | 0.942 | 0.71248 | 0.00038 | 0.923 | 0.948 | 0.11042 | 0.00016 | 0.938 | 0.885 | 1.00000 | 0.00000 |
| Clin_870 | 0.876 | 0.948 | 0.08264 | 0.00043 | 0.663 | 0.947 | 0.00000* | 0.00000 | 0.813 | 0.954 | 0.00024* | 0.00005 |
| Clin_4572 | 0.482 | 0.923 | 0.00000* | 0.00000 | 0.532 | 0.928 | 0.00000* | 0.00000 | 0.357 | 0.921 | 0.00000* | 0.00000 |
| Clin_23074 | 0.761 | 0.800 | 0.04081 | 0.00033 | 0.675 | 0.820 | 0.25159 | 0.00125 | 0.813 | 0.825 | 0.94119 | 0.00074 |
| Clin_33986 | 0.752 | 0.957 | 0.00000* | 0.00000 | 0.640 | 0.961 | 0.00000* | 0.00000 | 0.500 | 0.931 | 0.00000* | 0.00000 |
| Clin_50973 | 0.908 | 0.929 | 0.78673 | 0.00061 | 0.838 | 0.931 | 0.00000* | 0.00000 | 0.875 | 0.915 | 0.53761 | 0.00066 |
| **Mean** | 0.817 | 0.922 | 0.35216 | 0.00037 | 0.764 | 0.929 | 0.16034 | 0.00032 | 0.755 | 0.916 | 0.31966 | 0.00023 |
| **Number of Samples** | 121 | | | | 82 | | | | 16 | | | |

c)

|  | **Locality Group 1** | | | | **Locality Group 2** | | | | **Locality Group 3** | | | |
| --- | --- | --- | --- | --- | --- | --- | --- | --- | --- | --- | --- | --- |
| **Locus** | ***H_O_.*** | ***H_E_.*** | ***P_HW_*** | **s.d.** | ***H_O_.*** | ***H_E_.*** | ***P_HW_*** | **s.d.** | ***H_O_.*** | ***H_E_.*** | ***P_HW_*** | **s.d.** |
| Mamb_12420 | 1.000 | 0.821 | 1.00000 | 0.00000 | 0.471 | 0.456 | 0.75557 | 0.00093 | Locus is monomorphic: no test done | | | |
| Mamb_13883 | 0.500 | 0.464 | 1.00000 | 0.00000 | 0.765 | 0.747 | 0.71861 | 0.00135 | 0.846 | 0.618 | 0.08667 | 0.00084 |
| Mamb_8314 | 0.500 | 0.464 | 1.00000 | 0.00000 | 0.706 | 0.706 | 0.14672 | 0.00090 | 0.923 | 0.668 | 0.05545 | 0.00060 |
| Mamb_11469 | 0.250 | 0.250 | 1.00000 | 0.00000 | 0.412 | 0.772 | 0.00000* | 0.00000 | 0.462 | 0.745 | 0.10080 | 0.00026 |
| Mamb_15504 | 0.500 | 0.464 | 1.00000 | 0.00000 | 0.765 | 0.807 | 0.50650 | 0.00118 | 0.923 | 0.806 | 0.80301 | 0.00128 |
| Mamb_2892 | 0.000 | 0.857 | 0.00994 | 0.00024 | 0.882 | 0.959 | 0.39194 | 0.00026 | 0.769 | 0.957 | 0.02960 | 0.00021 |
| Mamb_3079 | 0.000 | 0.667 | 0.33366 | 0.00156 | 0.529 | 0.556 | 1.00000 | 0.00000 | 0.538 | 0.548 | 1.00000 | 0.00000 |
| Mamb_2411 | 1.000 | 0.857 | 0.66437 | 0.00136 | 0.941 | 0.954 | 0.11516 | 0.00029 | 0.846 | 0.929 | 0.20098 | 0.00038 |
| Mamb_9283 | 0.750 | 0.821 | 0.32023 | 0.00139 | 0.882 | 0.932 | 0.69811 | 0.00050 | 0.923 | 0.932 | 0.88502 | 0.00052 |
| Mamb_4540 | 1.000 | 0.929 | 1.00000 | 0.00000 | 0.882 | 0.891 | 0.69599 | 0.00093 | 0.615 | 0.852 | 0.01083 | 0.00014 |
| **Mean** | 0.550 | 0.660 | 0.73282 | 0.00046 | 0.724 | 0.778 | 0.50286 | 0.00063 | 0.761 | 0.784 | 0.35248 | 0.00047 |
| **Number of Samples** | 4 (*M. ambreensis*) | | | | 17 | | | | 13 | | | |
|  | **Locality Group 4** | | | |  |  |  |  |  |  |  |  |
| **Locus** | ***H_O_.*** | ***H_E_.*** | ***P_HW_*** | **s.d.** |  |  |  |  |  |  |  |  |
| Mamb_12420 | 0.286 | 0.495 | 0.15429 | 0.00100 |  |  |  |  |  |  |  |  |
| Mamb_13883 | 0.571 | 0.846 | 0.05423 | 0.00067 |  |  |  |  |  |  |  |  |
| Mamb_8314 | 0.714 | 0.725 | 0.87337 | 0.00090 |  |  |  |  |  |  |  |  |
| Mamb_11469 | 0.286 | 0.626 | 0.03434 | 0.00071 |  |  |  |  |  |  |  |  |
| Mamb_15504 | 0.429 | 0.681 | 0.06792 | 0.00070 |  |  |  |  |  |  |  |  |
| Mamb_2892 | 0.857 | 0.978 | 0.21327 | 0.00022 |  |  |  |  |  |  |  |  |
| Mamb_3079 | 0.667 | 0.485 | 1.00000 | 0.00000 |  |  |  |  |  |  |  |  |
| Mamb_2411 | 0.857 | 0.901 | 0.72247 | 0.00117 |  |  |  |  |  |  |  |  |
| Mamb_9283 | 0.714 | 0.956 | 0.04760 | 0.00037 |  |  |  |  |  |  |  |  |
| Mamb_4540 | 0.857 | 0.879 | 0.87035 | 0.00065 |  |  |  |  |  |  |  |  |
| **Mean** | 0.624 | 0.757 | 0.40378 | 0.00064 |  |  |  |  |  |  |  |  |
| **Number of Samples** | 7 (including two *M. ambreensis)* | | | |  |  |  |  |  |  |  |  |

d)

|  | **Locality Group 1** | | | | **Locality Group 2** | | | | **Locality Group 3** | | | |
| --- | --- | --- | --- | --- | --- | --- | --- | --- | --- | --- | --- | --- |
| **Locus** | ***H_O_.*** | ***H_E_.*** | ***P_HW_*** | **s.d.** | ***H_O_.*** | ***H_E_.*** | ***P_HW_*** | **s.d.** | ***H_O_.*** | ***H_E_.*** | ***P_HW_*** | **s.d.** |
| Mbell_158602 | 1.000 | 0.926 | 0.88027 | 0.00071 | 0.929 | 0.913 | 0.63516 | 0.00063 | 0.924 | 0.928 | 0.14179 | 0.00068 |
| Mbell_27880 | 0.364 | 0.476 | 0.35736 | 0.00192 | 0.250 | 0.741 | 0.00000* | 0.00000 | 0.093 | 0.505 | 0.00000* | 0.00000 |
| Mbell_16089 | 0.462 | 0.849 | 0.00000* | 0.00000 | 0.545 | 0.889 | 0.00000* | 0.00000 | 0.365 | 0.904 | 0.00000* | 0.00000 |
| Mbell_31964 | 0.200 | 0.926 | 0.00000* | 0.00000 | 0.378 | 0.918 | 0.00000* | 0.00000 | 0.352 | 0.939 | 0.00000* | 0.00000 |
| Mbell_102671 | 0.769 | 0.889 | 0.25895 | 0.00059 | 0.705 | 0.874 | 0.00085* | 0.00008 | 0.662 | 0.794 | 0.00000* | 0.00000 |
| Mbell_19325 | 0.222 | 0.850 | 0.00000* | 0.00000 | 0.225 | 0.878 | 0.00000* | 0.00000 | 0.224 | 0.846 | 0.00000* | 0.00000 |
| Mbell_333 | 0.846 | 0.926 | 0.04530 | 0.00046 | 0.795 | 0.926 | 0.01985 | 0.00009 | 0.939 | 0.938 | 0.02305 | 0.00014 |
| **Mean** | 0.552 | 0.835 | 0.22027 | 0.00053 | 0.547 | 0.877 | 0.09369 | 0.00011 | 0.508 | 0.836 | 0.02355 | 0.00012 |
| **Number of samples** | 13 | | | | 44 | | | | 66 | | | |
|  | **Locality Group 4** | | | | **Locality Group 5** | | | | **Locality Group 6** | | | |
| **Locus** | ***H_O_.*** | ***H_E_.*** | ***P_HW_*** | **s.d.** | ***H_O_.*** | ***H_E_.*** | ***P_HW_*** | **s.d.** | ***H_O_.*** | ***H_E_.*** | ***P_HW_*** | **s.d.** |
| Mbell_158602 | 0.692 | 0.730 | 0.45472 | 0.00115 | 0.733 | 0.703 | 0.03515 | 0.00062 | 0.855 | 0.890 | 0.82326 | 0.00056 |
| Mbell_27880 | 0.810 | 0.545 | 0.02531 | 0.00040 | 0.097 | 0.154 | 0.02221 | 0.00046 | 0.564 | 0.705 | 0.00000* | 0.00000 |
| Mbell_16089 | 0.269 | 0.554 | 0.00209 | 0.00015 | 0.903 | 0.705 | 0.04702 | 0.00044 | 0.691 | 0.790 | 0.10194 | 0.00046 |
| Mbell_31964 | 0.222 | 0.454 | 0.00062* | 0.00008 | 0.091 | 0.406 | 0.00063* | 0.00008 | 0.233 | 0.776 | 0.00000* | 0.00000 |
| Mbell_102671 | 0.185 | 0.171 | 1.00000 | 0.00000 | 0.581 | 0.688 | 0.08516 | 0.00047 | 0.745 | 0.889 | 0.00711 | 0.00014 |
| Mbell_19325 | 0.182 | 0.648 | 0.00000* | 0.00000 | 0.400 | 0.664 | 0.00000* | 0.00000 | 0.116 | 0.656 | 0.00000* | 0.00000 |
| Mbell_333 | 0.769 | 0.677 | 0.94497 | 0.00072 | 0.871 | 0.801 | 0.00000* | 0.00000 | 0.889 | 0.880 | 0.27355 | 0.00112 |
| **Mean** | 0.447 | 0.540 | 0.34682 | 0.00036 | 0.525 | 0.589 | 0.02717 | 0.00030 | 0.585 | 0.798 | 0.17227 | 0.00033 |
| **Number of samples** | 27 | | | | 31 | | | | 55 | | | |

Table S3. Pairwise F_ST_ values between populations (locality groups) of *B. tuberculata*, *C. linotum*, *M. ambony* / *M*. *ambreensis*, and *M. bellyi* calculated from 13, 8, 10, and 7 microsatellite loci with < 5 % missing data respectively. Significant values are italicized.

| ***B. tuberculata*** | **1** | **2** | **3** |  | | |  | | | |  |
| --- | --- | --- | --- | --- | --- | --- | --- | --- | --- | --- | --- |
| **1 Locality Group 1** | 0.00000 |  |  |  | | |  | | | |  |
| **2 Locality Group 2** | 0.05018 | 0.00000 |  |  | | |  | | | |  |
| **3 Locality Group 3** | 0.05627 | *0.03810* | 0.00000 |  | | |  | | | |  |
| ***C. linotum*** | **1** | **2** | **3** |  | |  | | | |  | |
| **1 Locality Group 1** | 0.00000 |  |  |  | |  | | | |  | |
| **2 Locality Group 2** | *0.00353* | 0.00000 |  |  | |  | | | |  | |
| **3 Locality Group 3** | *0.00116* | *-0.00231* | 0.00000 |  | |  | | | |  | |
| ***M. ambony / M. ambreensis*** | **1** | **2** | **3** | | **4** | | | |  | |  |
| **1 Locality Group 1** | 0.00000 |  |  | |  | | | |  | |  |
| **2 Locality Group 2** | 0.19562 | 0.00000 |  | |  | | | |  | |  |
| **3 Locality Group 3** | 0.24993 | *0.02370* | 0.00000 | |  | | | |  | |  |
| **4 Locality Group 4** | 0.14451 | *0.01758* | *0.03601* | | 0.00000 | | | |  | |  |
| ***M. bellyi*** | **1** | **2** | **3** | | **4** | | | **5** | | | **6** |
| **1 Locality Group 1** | 0.00000 |  |  | |  | | |  | | |  |
| **2 Locality Group 2** | *0.00886* | 0.00000 |  | |  | | |  | | |  |
| **3 Locality Group 3** | *0.02006* | *0.02155* | 0.00000 | |  | | |  | | |  |
| **4 Locality Group 4** | 0.22137 | 0.17987 | 0.19337 | | 0.00000 | | |  | | |  |
| **5 Locality Group 5** | 0.13773 | 0.11970 | 0.13724 | | 0.32901 | | | 0.00000 | | |  |
| **6 Locality Group 6** | 0.05105 | *0.04154* | 0.05820 | | 0.16475 | | | 0.12105 | | | 0.00000 |
